# Stain-free artificial intelligence-assisted light microscopy for the identification of leukocyte morphology change in presence of bacteria

**DOI:** 10.3389/fbinf.2025.1725145

**Published:** 2026-01-06

**Authors:** Alexander Hunt, Holger Schulze, Kay Samuel, Robert B. Fisher, Till T. Bachmann

**Affiliations:** 1 Centre for Inflammation Research, Institute for Regeneration and Repair, The University of Edinburgh, Edinburgh, United Kingdom; 2 Tissues, Cells and Advanced Therapeutics, Scottish National Blood Transfusion Service, NHS National Services Scotland, Edinburgh, United Kingdom; 3 School of Informatics, The University of Edinburgh, Edinburgh, United Kingdom

**Keywords:** artificial neural network, morphological analysis, YOLOv4, blood analysis, leukocyte

## Abstract

**Background:**

Rapid detection of bacterial infections through leukocyte activation analysis could significantly reduce diagnostic timeframes from days to hours. Traditional methods like flow cytometry and biomarker assays face limitations including technical complexity, equipment requirements, and delayed results.

**Methods:**

We developed a dual artificial neural network system combining stain-free light microscopy with microfluidic technology to detect morphological changes in activated leukocytes. YOLOv4 networks were trained using five-fold cross-validation on images of chemically stimulated leukocyte subpopulations (lymphocytes, monocytes, and neutrophils) and validated against flow cytometry. The system was tested on whole blood samples spiked with *E. coli* at clinically relevant concentrations (10–250 CFU/mL).

**Results:**

The optimized four-class network achieved high performance metrics for lymphocytes (F1 score: 80.1% ± 2.5%) and neutrophils (F1 score: 91.7% ± 1.7%), while a specialized binary classifier for monocytes achieved 97.0% ± 2.8% F1 score. In bacteria-spiked whole blood experiments, the system successfully detected activated leukocytes within 30 min at concentrations approaching clinical blood culture detection limits (11.11 ± 4.79 CFU/mL). Neutrophils showed rapid activation peaking at 1–3 h, while lymphocyte activation increased gradually over 6–12 h, consistent with innate versus adaptive immune response kinetics.

**Conclusion:**

This AI-assisted microscopy platform enables rapid, label-free detection of leukocyte activation in response to bacterial infection with minimal sample handling and no requirement for specialized staining or trained technicians. The technology demonstrates potential for accelerating infection diagnosis and could be extended to other pathologies with morphological manifestations.

## Introduction

The ability to accurately detect and characterise activated white blood cells is of paramount importance in both clinical and research settings. Activated leukocytes play a crucial role in the human immune system, constituting the fundamental basis of immunological responses, functioning as guards against pathogenic invasion, mediators of inflammation, and coordinators of the immunological cascade (Nedeva, 2021). Detecting white cell activity has become a promising technique among the many ways to provide information about the inflammatory response to infection. Conventional approaches often rely on biomarker tests, such as C-reactive protein (CRP) and Procalcitonin (PCT), which, while effective, can sometimes lack specificity and speed (Samsudin and Vasikaran, 2017). To improve patient care and support broader public health initiatives, this paper focuses on sophisticated approaches for identifying white cell activation, including flow cytometry-based immunophenotyping, quantitative cell surface marker analysis, and analysis of activation morphology (Kapellos et al., 2019; Robinson et al., 2023). These advanced analytical methods can significantly enhance both the accuracy and timeliness of infection diagnosis by detecting cellular activation patterns that often precede clinical manifestations, potentially reducing diagnostic timeframes from days to hours (Opota et al., 2015). A comprehensive understanding of leukocyte activation status and behaviour provides critical insights for diagnosing and monitoring various pathological conditions, including acute bacterial infections, autoimmune disorders, and haematological malignancies (Chen et al., 2017; Nedeva, 2021; Döhner et al., 2022). While several established techniques exist for characterising white cell activation, each with unique strengths and applications in specific clinical contexts, it is essential to acknowledge their inherent limitations, including technical complexity, equipment requirements, and interpretive challenges that may restrict their broader implementation in resource-limited settings.

Flow cytometry emerged as a powerful tool for multiparametric analysis, enabling the simultaneous detection of multiple activation markers with high sensitivity and specificity (O’Donnell et al., 2013). By employing fluorescently labelled antibodies and light scattering principles, flow cytometry provided quantitative insights into the activation status of white blood cell populations (Robinson et al., 2023). Clinicians use flow cytometry to diagnose a variety of conditions, including haematological cancers like leukaemia and lymphoma that can be precisely tracked through changes in lymphocyte marker expressions, assess immune deficiencies by detailed cellular characterisation, and monitor chronic diseases like HIV by tracking immune cell populations and their functional states (Thiel, 1985; McSharry et al., 1990; Kanegane et al., 2018). Researchers can identify specific leukocyte subtypes using surface markers such as CD45 (Leukocytes), CD3 (T lymphocytes), CD19 (B lymphocytes), and CD56 (NK cells), while simultaneously measuring cell size, internal complexity, and protein expression patterns (Liu et al., 2020; Rustam et al., 2022; Rahman and Bordoni, 2024). However, flow cytometry has several drawbacks. It requires specialised equipment and trained personnel and can be costly. Sample preparation is extensive and time-consuming, and there is a risk of cell damage during the process, potentially affecting results (Sazonova et al., 2022; Robinson et al., 2023).

ELISAs complemented flow cytometry by enabling the quantification of soluble markers of cell activation, such as cytokines or secreted proteins. The high specificity of ELISAs stems from their unique ability to detect and quantify molecular targets with remarkable precision using highly specific antibody-antigen interactions (Waritani et al., 2017). Antibodies are carefully selected to recognise distinct epitopes, enabling researchers to differentiate between closely related molecules with minimal cross-reactivity. This specificity is further enhanced by multi-step validation processes, including the use of blocking agents and stringent washing protocols that eliminate non-specific binding, thereby reducing false-positive and false-negative results.

In infectious disease research, ELISA specificity becomes particularly critical for distinguishing pathogen-specific immune responses. For instance, in SARS-CoV-2 studies, researchers have developed targeted assays that detect specific viral proteins, such as nucleocapsid antigens, with up to 99.3% specificity (Luo et al., 2022). The technique’s molecular discrimination allows for precise identification of antibodies against pathogens, even in complex biological samples containing multiple potential interfering molecules, making ELISAs an invaluable tool for understanding host-pathogen interactions and immune recognition. However, ELISA may not fully capture the complexity of the activation process. Precise sample handling is crucial to avoid contamination and ensure accurate results (Waritani et al., 2017).

Immunohistochemistry (IHC) offers a unique perspective by preserving the spatial context of activated white blood cells within tissue architecture. This technique allowed for the visualisation of activation marker expression *in situ*, facilitating a better understanding of the localisation and interactions of these cells within their native microenvironments (Tan et al., 2020). The interpretation of staining can be subjective and vary between observers. Technical variability in tissue processing and staining protocols can impact results, and IHC generally provides qualitative or semi-quantitative data rather than precise quantification (O’Hurley et al., 2014).

Western blotting proved valuable for the specific detection and relative quantification of activation-associated proteins, providing complementary information to gene expression analyses (Mishra et al., 2017). By separating and probing proteins based on their molecular weight, Western blotting offered insights into the expression levels and isoforms of activation markers, contributing to a more comprehensive characterisation of the activation state. However, it is a labour-intensive technique that offers semi-quantitative data and requires relatively large amounts of protein, which may not be available in all samples (Mahmood and Yang, 2012).

Reverse transcription PCR (RT-PCR) and quantitative PCR (qPCR) techniques enabled the sensitive and specific detection of activation marker gene expression at the mRNA level (Ho-Pun-Cheung et al., 2009; Kuang et al., 2018). These methods were particularly useful for identifying early transcriptional changes associated with cell activation and for studying the regulatory mechanisms underlying the activation process. That said, RNA is less stable than DNA, making sample handling critical to avoid degradation (Opitz et al., 2010; Marshall et al., 2021). These techniques also require expertise in molecular biology and can be expensive due to the reagents and equipment needed.

Microscopy-based techniques, such as fluorescence microscopy, allowed for the high-resolution visualisation of activated white blood cells, providing insights into their morphology, marker expression patterns, and spatial relationships within the sample (Hickey et al., 2021). These methods were invaluable for studying cell-cell interactions, localisation, and the dynamics of activation processes. They may not be able to resolve very fine details depending on the microscopy technique used. This can be mitigated in super-resolution fluorescence microscopy, however, requiring more complex equipment (Valli and Sanderson, 2021). Additionally, these methods generally provide qualitative data and are less effective for quantitative analysis, often requiring manual counting and analysis, which can be labour-intensive and prone to human error, which can be mitigated using algorithms (Sailem et al., 2016; Shanmugam et al., 2018).

The combination of these techniques provides a comprehensive toolbox for investigating activated white blood cells from various angles, each contributing unique insights and complementing the others. Integrating the data obtained from these methods allows for a more holistic understanding of the activation processes, enabling the development of novel diagnostic and therapeutic approaches and advancing the fundamental knowledge of the immune system.

As these research avenues converge, a more comprehensive and nuanced understanding of immune cell activation is expected to emerge, paving the way for improved diagnostic capabilities, more targeted therapeutic interventions, and a deeper appreciation of the immune system’s intricate workings in both health and disease. Combining light microscopy, artificial intelligence and microfluidics, the sample handling is minimal, thus reducing some of the issues that can arise from sample preparation. Using microscopy, while it can lose information on the finer features, having a neural network analyse the data removes the possibility of human observer bias standardising the feature detection. Additionally, post-analysis can also be applied to the images, thereby inferring further results without having to prepare the sample for another technique. Additionally, integrating AI with microscopy removes the need for expensive equipment and software where the analysis is already provided at the output. Furthermore, the integration of single-cell analysis techniques into population analysis promises to unveil unprecedented insights into the heterogeneity of activated cell populations, facilitating the detection and analysis of activated white cells within a heterogeneous population. To achieve this, the ability to train artificial neural networks to discriminate between activated and unstimulated leukocytes was investigated using isolated subpopulations and verified in whole blood by comparison with standard haematological techniques such as fluorescence flow cytometry.

## Materials and methods

### Blood sample preparation

24 10 mL blood samples from healthy donors at the CIR Blood Bank (CIR—21-EMREC-041) were collected and prepared in one of two ways, depending on the experiment: either for direct sample analysis or for separation of cell types for training.

#### Direct observation

A 100 µL sample of human whole blood collected from donors was diluted into 1900 µL of diluent (PBS pH 7.2 + 5% EDTA). The sample was then loaded into a BD Plastipak 10 mL syringe with a Luer Lok connector, connected to the system described in our previous publication (Hunt et al., 2025), and observed under flow conditions (20 min at 25 μL.min^−1^). This equates to about 12,000 frames recorded for analysis (at a frame rate of 10 frames recorded per second).

#### Sample preparation for training

Different treatments were applied to the cells depending on the desired training material needing to be imaged. Whole blood cells were separated into different sub-populations of cells using density gradient separation technique (Hunt et al., 2025). Whole blood were diluted 1:1 with PBS and layered over Ficoll-Paque Plus (GE17-1440-02, Sigma Aldrich) before centrifugation at 450 × g for 35 min (medium acceleration and brake). The mononuclear cell layer (monocytes and lymphocytes) was collected, washed twice in PBS (350 × g, 5 min), and resuspended in RPMI-1640 supplemented with 10% FBS. After 2 h incubation at 37 °C, non-adherent lymphocytes were harvested, while adherent monocytes were detached using TrypLE Express (10 min, 37 °C), collected, and washed.

The remaining pellet, containing erythrocytes and neutrophils, was washed in PBS and treated with red blood cell lysis buffer (BioLegend) for 7 min at room temperature. Neutrophilic cells were recovered by centrifugation (350 × g, 7 min) and washed in PBS. If erythrocyte contamination persisted, lysis was repeated for 3 min. All fractions were resuspended in PBS (pH 7.4) or RPMI + 10% FBS for downstream applications.

To obtain stimulated populations of different white cell types (lymphocytes, monocytes and neutrophils), each subpopulation was isolated from one another and seeded to 5 × 10^5^ cells per flask (as described in Hunt et al., 2025). Cells were cultured in RPMI + 10% FBS alone or with the addition of specific stimulatory molecules. For lymphocyte: 1 5 μg.mL^−1^ phytohaemagglutinin + 10 ng.mL^−1^ IL-2 (PHA; ThermoFisher, Waltham, USA: IL-2 PeproTech, Cranbury, NJ, USA) and 4 μg.mL^−1^, concanavalin A (Con A; ThermoFisher, Waltham, USA). IL-2 is required as it helps activate lymphocytes (Ross and Cantrell, 2018; O’Donovan et al., 1995; Ando et al., 2014). For monocytes: 0.1 μg.mL^−1^ lipopolysaccharide (LPS; ThermoFisher, Waltham, USA). For neutrophils 43.8 μg.mL^−1^ N-formylmethionyl-leucyl-phenylalanine (fMLP; Sigma Aldrich, St. Louis, MI, USA). Stimulated cells were incubated for 16 h to ensure maximum stimulation. Stimulation was verified using flow cytometry, where activation-induced changes in cell morphology were confirmed through forward and side scatter profiles shift. Following verification of successful stimulation, leukocytes were introduced into the microfluidic system, one population at a time. This approach enabled the consistent acquisition of images of activated and non-activated leukocytes in each sub-population for neural network training.

All input images were resized to 416 × 416 pixels, consistent with YOLOv4’s native input dimension requirements. Images were normalised by scaling pixel intensity values between 0 and 1 and stored in RGB JPEG format without compression artifacts. No colour correction or histogram equalisation was applied to preserve the original microscopy intensity distribution. These augmentations were implemented using custom Python 3 scripts in conjunction with the OpenCV and augmentations libraries to automate augmentation pipelines. Each transformation was applied randomly with a 0.3 probability per image to avoid overfitting to synthetic transformations. The inclusion of augmented images increased the dataset size by approximately sixfold, resulting in improved model generalization as evidenced by reduced validation loss variance across folds (<5%) and higher recall for minority classes such as neutrophils and platelets.

#### Plasma thawing and freezing

Plasma samples (from human whole blood) containing various EDTA anticoagulants were stored to preserve haemostatic function and prevent inadvertent coagulation at −80C until thawing. Plasma and RPMI + 10% plasma were used as a media for white cell incubation with bacteria and tests for bacterial growth kinetics.

A water bath was preheated to 37 °C. Frozen plasma aliquots were removed from −80 °C storage and immediately immersed in the 37 °C water bath. Samples were gently agitated at 300 revolutions per minute to promote uniform thawing. The total thawing time was 45 min. Once completely thawed, samples were used immediately or stored at 4 °C for up to 24 h until use or used immediately. Storage below 0 °C was avoided to prevent cold-induced coagulation.

### Bacterial preparation

#### Liquid culture

A sterile lysogeny broth (LB, ThermoFisher, Waltham, USA) medium was utilised to create a bacterial culture of *E. coli* isolated from a clinical isolate from a urine sample collected at the Royal Infirmary of Edinburgh. Using aseptic techniques, 5 mL of medium was inoculated with a colony obtained from a plate culture. The liquid culture was incubated in a shaking incubator overnight (18 h) at 37 °C, shaking at a rate of 150 revolutions per minute. Following incubation, the broth was turbid, indicating bacterial growth. A subculture was performed overnight to remove debris and stimulate the bacteria. After incubation, the optical density of the culture at 600 nm was measured using a Cary 60 UV-Vis spectrophotometer (Agilent Technologies, Santa Clara, CA, USA), and an approximate colony-forming unit per mL was calculated using the equation below (Couto et al., 2018):

$${OD}_{600}*8\times 10^{8}=CFU.{mL}^{-1}$$

The equation was used as a starting point to prepare bacterial dilutions for plate counts. The liquid culture was then diluted into PBS pH 7.4 to achieve the desired concentration of bacteria.

#### Plate culture

A plate culture was prepared by plating liquid Oxoid nutrient agar (ThermoFisher, Waltham, USA) into sterile Petri dishes. Once the agar had solidified, a swab was used to streak bacteria from liquid culture, glycerol stock, or sample onto the dish. After overnight incubation at 37 °C, colonies appear on the agar plates. Individual colonies can be swabbed and placed into liquid cultures for further experimentation.

#### Bacterial growth curve

The optical density at 600 nm is measured using liquid cultures as a starting point. The culture was diluted into Oxoid media, giving an OD_600_ = 0.01, then loaded into a flat-bottomed 96-well plate kept at 37 °C and OD_600_ read every 20 min using a Biotek Synergy HT plate reader (Agilent Technologies, Santa Clara, CA, USA). The plate was shaken between measurements. Data was analysed by deducting the density of the control well (media alone) from the wells with bacteria to calculate the density attributed to the growth of the bacteria and no other components of the media. Bacterial plate counts were used to verify the CFU seeding concentration of bacteria in concentration-dependent experiments.

#### Bacterial plate counting

Bacterial enumeration was performed using a standard plate counting technique. Oxoid nutrient agar plates were prepared by suspending nutrient agar powder in distilled water. The mixture was then autoclaved, and once cooled to approximately 50 °C, the molten agar was poured into sterile Petri dishes (Fisherbrand, Waltham, MA, USA). The plates were allowed to solidify at room temperature before use. Bacterial samples were serially diluted 1:10 in PBS (pH 7.2) to obtain countable CFU densities. From each dilution, 100 μL volumes were pipetted onto the surfaces of the triplicate pre-poured nutrient agar plates and allowed to dry. Inoculated plates were incubated in an inverted position at 37 °C for 18–24 h to allow colony formation.

Following incubation, colonies were counted manually. CFU/mL values for the original samples were calculated based on the dilution factors. All plating work was conducted using aseptic techniques. Positive (*E. coli*) and negative (uninoculated media) control plates were included in each experiment.

### Flow cytometry

Sample preparation and data acquisition followed the same process as in Hunt et al., (2025). Population counts were extracted from FlowJo and plotted in GraphPad Prism. For plots, the populations were juxtaposed on one scatter plot in FlowJo and exported to GraphPad Prism to arrange the results (gating strategy in Supplementary Appendix).

### Data pipeline

A dual deep learning pipeline was developed to classify and quantify leukocytes in stain-free brightfield microfluidic images. The system builds upon a pretrained YOLOv4 model previously optimized for whole-blood leukocyte differentials (Hunt et al., 2025). The rationale for transfer learning was to leverage established feature representations of unstained blood cell morphology, reducing the amount of task-specific data and computational time required compared to training a model from scratch. Preliminary experiments confirmed that retraining from scratch using the present dataset led to overfitting and lower performance, supporting the use of a pretrained backbone for efficient and generalizable feature extraction.

Model training and inference were performed in Python using the Darknet framework with CUDA acceleration. The computer used is an HP ELITEDESK equipped with an Intel CoreTM i5-6500 CPU (Intel, Santa Clara, CA, USA), 16 Gb of RAM and an NVIDIA RTX A2000 12GB GPU (Nvidia, Santa Clara, CA, USA). On the server, the server-side program can either be run locally on UNIX (here, ubuntu 20.04 LTS, Canonical, London, UK) or on a Docker container (Docker, Inc., Palo Alto, CA, USA). Initially, the UNIX version was used during testing; once a stable version was coded, the program was moved to the Docker version. The Docker implementation is built on NVIDIA CUDA 11.7.1 with Ubuntu 20.04 as the base system, configured with CUDNN 8 acceleration and OpenCV 4.6.0 integration. Input images were exported directly from the microfluidic imaging system as uncompressed JPEGs, with a mean field of view of approximately 57 × 57 µm. Each image was resized to 416 × 416 pixels to match the YOLOv4 input requirements, while preserving aspect ratio through zero-padding. No additional colour normalization was required, as all images were captured under uniform illumination and exposure conditions. To mitigate class imbalance, minority classes were augmented more extensively (rotation, flip, brightness/contrast variation). Class weights inversely proportional to frequency were applied in the loss function. Together inference time took between 10–15 min depending on the dataset size.

Data augmentation consisted of deterministic geometric and intensity-based transformations implemented within the Darknet training pipeline. Augmentations included random rotations (±90°), flips, and brightness or contrast scaling to improve model robustness to cell orientation and imaging variation. These were applied uniformly across all training epochs, ensuring consistent augmentation coverage without altering class balance.

Using the pre-trained whole blood differential network trained on unstimulated freshly isolated blood samples, the artificial neural network pipeline for distinguishing the presence of activated white cells, the new models were trained on YOLOv4 using five-fold cross-validation (donor images were pooled, the pool was subsequently split into five datasets randomly shuffled). Those five subsets were then redistributed randomly five times into three sets: three sets pooled for training, one for validation and one for testing. This allowed statistical analysis with standard deviation of the acquired data. For each training regimen, the original image data was split into independent cropped images, each with an independently detected single cell ensuring parts of the original uncropped image are within the training, testing and validation sets. The network was trained using the following hyperparameters: a batch size of 64 with 64 subdivisions, and input images were 416 × 416 pixels with three-channels. We used a momentum of 0.949 and an L2 weight decay of 0.0005. The initial learning rate was to 1 × 10^−4^, with a burn-in period of 1,000 iterations. Training proceeded for 10,000 iterations, with scheduled learning-rate reductions applied at 8,000 and 9,000 iterations.

Model performance was evaluated using standard object detection metrics: precision, recall, and F1-score at an intersection-over-union (IoU) threshold of 0.5. These metrics were chosen to capture both detection accuracy (localization and classification) and class-specific balance, which are critical in biomedical imaging contexts where both false positives and false negatives carry diagnostic implications. The evaluation was performed on a held-out test set comprising 20% of the total dataset, stratified by donor to avoid subject-specific bias.

**FIGURE 1:**
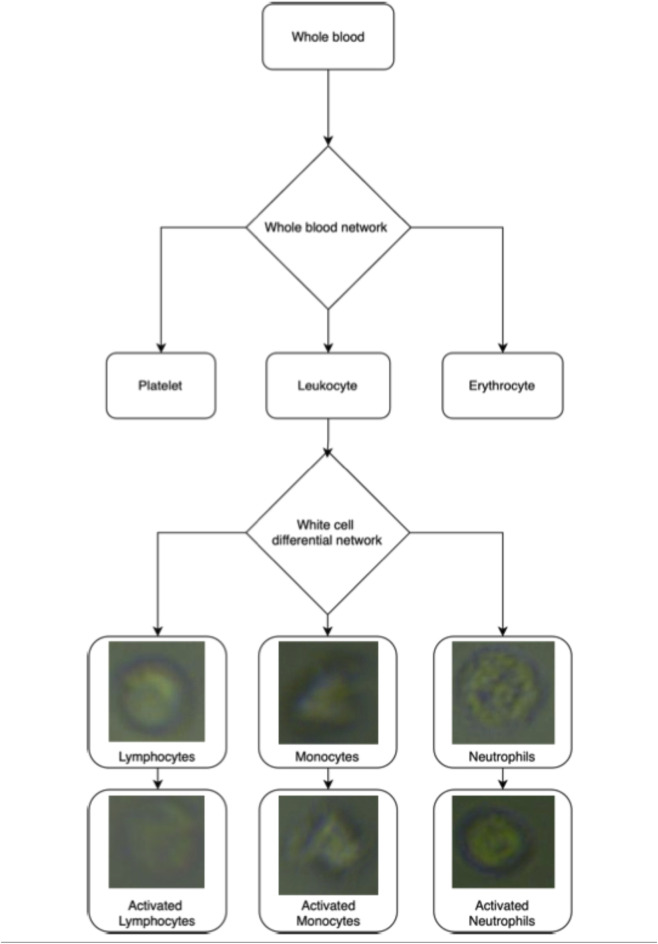
Artificial neural network pipeline for inferring leukocyte status from a full blood sample (cell images not to scale).

Following detection, a secondary neural network was used for post-classification morphological analysis to detect activation-associated changes within leukocyte subsets (Figure 1). Together, the two-stage AI system enables high-throughput, stain-free assessment of leukocyte morphology directly from microfluidic images. The full code repository is available at the following repository: https://github.com/alex1075/machine-code.git.

### Bacteria spiked whole blood tests

For whole blood bacterial seeding experiments, 5 mL of freshly collected peripheral blood was obtained from three healthy donors. Each sample was seeded with *E. coli* (clinical isolate from the Royal Infirmary of Edinburgh) at a concentration of around 10 CFU per ml (obtained through serial dilutions and verified with plate culture). The seeded blood samples were incubated at 37 °C with gentle agitation (50 rpm) for 2 h to allow leukocyte activation to occur. Following incubation, samples were imaged using the following the setup from Hunt et al., (2025). Eosinophils and basophils were not included in training owing to their low frequency (<2% of leukocytes). During inference, such cells were typically assigned low confidence scores (<0.3) and classified as ‘non-detected’. Their exclusion may marginally reduce specificity but is unlikely to affect the classification of dominant leukocyte types. Future dataset expansion will include these populations to improve robustness.

These images were processed using the dual neural network approach. Using previously published whole blood differential network first identified and isolated all leukocyte images, which were subsequently analysed by the specialised white cell differential networks described below (see Tables 3–6; Hunt et al., 2025).

## Results

### Inducing leukocyte activation

**FIGURE 2:**
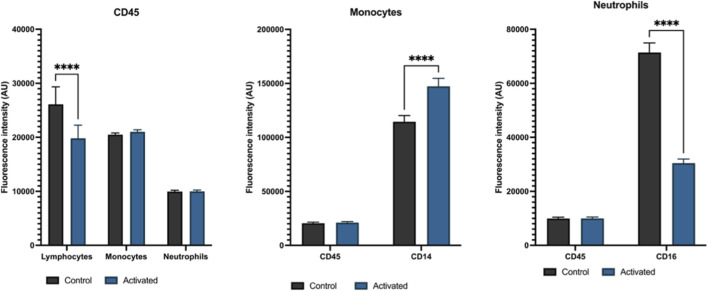
Plotted fluorescence change of activated leukocytes compared to their control counterparts. Each subtype was treated with a chemical or cocktail of chemicals known to trigger their activation. After a 16-hour incubation, each cell type was fixed and stained with fluorescent antibodies corresponding to the markers present on the surface of each subtype. Isolated subpopulations from three donors, n = 3 replicates per cell type. While both monocytes and neutrophils do not express much difference between their control and treated CD45 expression, their specific CD marker do change significantly (CD14 increases for monocytes, and CD16 decreases for neutrophils). CD45 expression for lymphocytes decreases, however, after activation. Two-way ANOVAs were performed (n = 3, p-CD45-lymphocytes <0.0001, p-CD14-monocytes <0.0001, p-CD16-neutrophils <0.0001).

From the literature, it has been established that different compounds can trigger the activation of leukocytes. Lymphocytes can be activated using phytohaemagglutinin (PHA) and concanavalin A (Con A); monocytes using lipopolysaccharides (LPS); neutrophils using N-formylmethionyl-leucyl-phenylalanine (fMLP) (O’Donovan et al., 1995; Ando et al., 2014). The effect of activation of treated and untreated leukocyte sub-populations with known concentrations of the relevant activator for 16 h (overnight) was verified by flow cytometry (Figure 2). The figure makes evident the effect the chemical treatment has had on the different leukocyte populations. The CD45 expression alone does not differ significantly between activated and non-activated monocytes and neutrophils. CD14 expression in monocytes increased after 16-h incubation with LPS, confirming that monocytes were activated. Whereas CD16 expression decreased after 16-h incubation with fMLP, suggesting neutrophils were exhausted after prolonged activation. Lymphocyte expression of CD45 expression appears not to increase on mitogen stimulation; this was expected due to the time constraint of the experiment.

### AI training on chemically induced leukocyte activation

**FIGURE 3:**
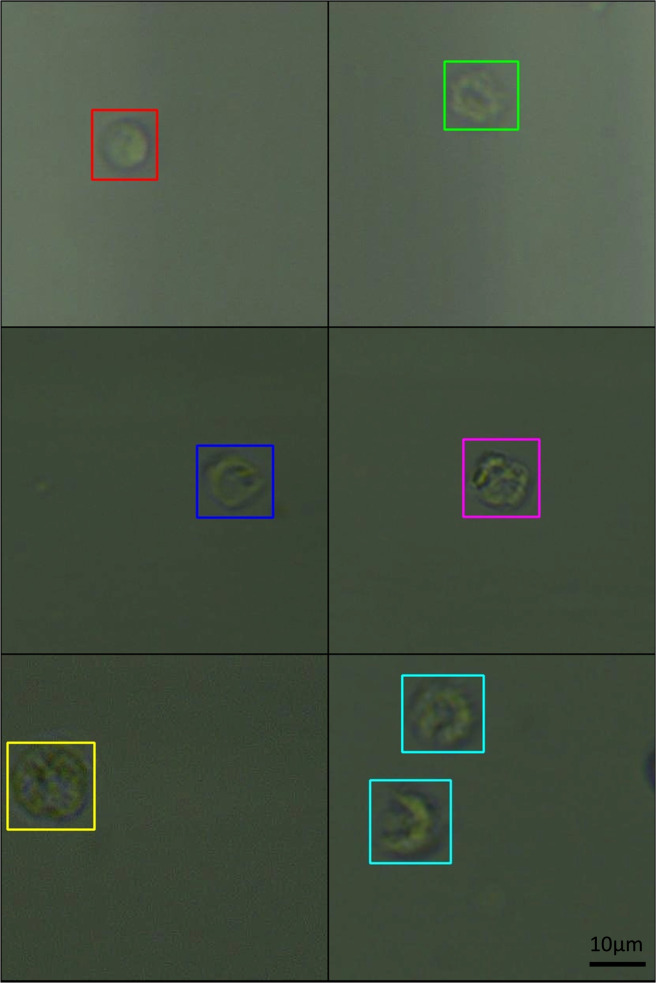
Combination of labelled images of leukocytes and their activated forms. Red box shows a lymphocyte with the green containing an activated lymphocyte. The blue box shows a monocyte with the magenta box containing an activated monocyte. The yellow box shows a neutrophil with the cyan boxes displaying activated neutrophils. The activation of white cells was achieved by incubating each population in Con A + PHA, LPS and fMLP respectively. The dimensions of the images are 416 × 416 pixels, or 57.37 × 57.37 µm. The cells were imaged at 40x zoom.

A dataset of images was collected for leukocytes after activation and those kept in control conditions (Figure 3). AI training was done using five-fold validation. After augmenting the dataset using 90, 180, and 270-degree rotation, 11,297 individual 416 × 416 images were generated. The model was trained on a dataset comprising 1,768 total original, unaugmented images across six cell classes: 390 lymphocytes, 491 activated lymphocytes, 317 neutrophils, 436 activated neutrophils, 69 monocytes, and 65 activated monocytes. Five-fold cross-validation was employed, with approximately 20% of the data held out for testing in each fold.

Tables 1, 2 shows that the YOLOv4 white cell differential model can discriminate between con A stimulated and unstimulated lymphocytes and fMLP stimulated and unstimulated neutrophils. Lymphocytes and their activated counterparts exhibit moderate classification performance, with precision values of 61.1% ± 3.0% and 54.7% ± 2.5% respectively. The recall metrics (56.2% ± 3.2% for lymphocytes and 58.5% ± 4.4% for activated lymphocytes) indicate that the model successfully identifies slightly more than half of these cells when present. This results in balanced F1 scores of 58.5% ± 1.9% and 56.5% ± 2.7% for the respective lymphocyte populations.

**TABLE 1:** Five-fold training outcome of stimulated leukocytes (lymphocytes, monocytes, and neutrophils) and their non-stimulated counterparts of a YOLOv4 network model. Detailed breakdown of the Precision, Recall and F1 score per blood cell subtype of the 5-fold cross validation of the binary network. Values are in percentage.

Cell type	Precision	Recall	F1 score
Lymphocytes	61.1 ± 3.0	56.2 ± 3.2	58.5 ± 1.9
Lymphocytes - activated	54.7 ± 2.5	58.5 ± 4.4	56.5 ± 2.7
Monocytes	5.2 ± 2.2	14.1 ± 9.4	7.4 ± 3.3
Monocytes - activated	6.67 ± 14.9	2.5 ± 5.6	3.6 ± 8.1
Neutrophils	61.1 ± 2.4	52.7 ± 3.6	56.5 ± 3.0
Neutrophils – activated	62.3 ± 2.4	52.7 ± 3.6	56.5 ± 3.0
Network	41.9 ± 2.8	41.2 ± 2.0	40.9 ± 1.8

**TABLE 2:** Five-fold training outcome of stimulated leukocytes (lymphocytes, monocytes, and neutrophils) and their non-stimulated counterparts of a YOLOv4 network model. Confusion matrix of one-fold or the 5-fold trained network of the YOLOv4 model trained to discriminate between lymphocytes, activated lymphocytes, monocytes, activated monocytes, neutrophils, and activated neutrophils.

		Prediction						
		Lymphocyte	Lymphocyte - activated	Monocyte	Monocyte - activated	Neutrophil	Neutrophil - activated	Non detected
Ground truth	Lymphocyte	212	107	6	5	24	36	0
	Lymphocyte – activated	75	320	8	1	28	49	10
	Monocyte	4	5	1	1	0	1	0
	Monocyte – activated	1	3	0	0	0	2	0
	Neutrophil	14	65	3	4	152	77	2
	Neutrophil – activated	20	95	8	0	59	253	1

For neutrophils and activated neutrophils similar performance to lymphocytes, with precision values of 61.1% ± 2.4% and 62.3% ± 2.4% respectively was seen. Both populations share identical recall rates of 52.7% ± 3.6%, yielding F1 scores of 56.5% ± 3.0% for both cell types. This suggests the model has similar capabilities in identifying neutrophils regardless of their activation status.

However, the model struggles significantly with monocyte classification. Non-activated monocytes exhibit extremely low precision (5.2% ± 2.2%) and recall (14.1% ± 9.4%), resulting in a poor F1 score of just 7.4% ± 3.3%. Activated monocytes fare even worse, with marginally higher precision (6.67% ± 14.9%) but substantially lower recall (2.5% ± 5.6%), culminating in an F1 score of only 3.6% ± 8.1%. These metrics highlight a limitation with the trained model’s ability to reliably detect and discriminate monocytes.

Overall, the network obtained a modest performance across every cell type, with combined metrics of 41.9 ± 2.8% precision, 41.2 ± 2. 0% recall, and 40. 9 ± 1. 8% F1 score. This moderate level of performance is mainly influenced by the acceptable classification of lymphocytes and neutrophils, while being significantly set back by inadequate monocyte detection.

To further inspect specific classification trends, the confusion matrix shown in Table 2 shows lymphocytes, where 212 were accurately classified, with 107 misclassified as activated lymphocytes and fewer misattributed to other cell types. Likewise, 320 activated lymphocytes were accurately identified, with 75 misclassified as non-activated. Neutrophils displayed a more intricate pattern, with 152 correctly classified but considerable misclassifications spread across activated neutrophils (77) and activated lymphocytes (65).

The model’s primary difficulty with monocytes is exemplified by the confusion matrix, which shows that only 5 of the 13 non-activated monocytes were correctly identified and only 3 of the 6 activated monocytes were properly classified. This is in line with the poor statistical metrics and highlights a major limitation in the model’s performance for these cell types.

Removing the monocytes and activated monocytes from the training data, a new version of the network was trained (Tables 3, 4). The new model demonstrates improved performance across all metrics compared to the previous six-class model (model detailed in Tables 1, 2). For lymphocytes, the precision reached 82.1% ± 2.1% (compared to 61.1% ± 3.0% previously), while recall improved to 78.3% ± 4.6% (previously 56.2% ± 3.2%). This resulted in an improved F1 score of 80.1% ± 2.5% (compared to 58.5% ± 1.9% previously). Similarly, activated lymphocytes also showed improvements, with precision of 79.8% ± 4.1% (up from 54.7% ± 2.5%), recall of 83.2% ± 1.5% (improved from 58.5% ± 4.4%), and an F1 score of 81.4% ± 2.3% (previously 56.5% ± 2.7%). Neutrophils and their activated counterparts showed more improvement. Non-activated neutrophils achieved a precision of 85.6% ± 1.5% (compared to 61.1% ± 2.4% previously) and recall of 79.4% ± 4.1% (up from 52.7% ± 3.6%), yielding an F1 score of 87.0% ± 2.5% (an improvement from 56.5% ± 3.0%). The best gain in performance was observed for activated neutrophils, with precision of 92.4% ± 1.1% (previously 62.3% ± 2.4%), recall of 91.0% ± 2.9% (up from 52.7% ± 3.6%), and an F1 score of 91.7% ± 1.7% (compared to 56.5% ± 3.0% previously).

**TABLE 3:** Filve-fold training outcome of stimulated lymphocytes and neutrophils only and their non-stimulated counterparts of a YOLOv4 network model. Detailed breakdown of the Precision, Recall and F1 score per blood cell subtype of the 5-fold cross validation of the binary network. Values are in percentage.

Cell type	Precision	Recall	F1 score
Lymphocytes	82.1 ± 2.1	78.3 ± 4.6	80.1 ± 2.5
Lymphocytes - activated	79.8 ± 4.1	83.2 ± 1.5	81.4 ± 2.3
Neutrophils	85.6 ± 1.5	79.4 ± 4.1	87.0 ± 2.5
Neutrophils – activated	92.4 ± 1.1	91.0 ± 2.9	91.7 ± 1.7
Network	87.5 ± 2.1	83.0 ± 2.0	82.0 ± 1.6

**TABLE 4:** Five-fold training outcome of stimulated lymphocytes and neutrophils only and their non-stimulated counterparts of a YOLOv4 network model. Confusion matrix of one fold of the five-fold cross-validated model of the YOLOv4 model trained to discriminate between lymphocytes, activated lymphocytes, neutrophils, and activated neutrophils.

		Prediction				
		Lymphocyte	Lymphocyte – activated	Neutrophil	Neutrophil - activate	Non detected
Ground truth	Lymphocyte	338	108	0	3	5
	Lymphocyte – activated	57	440	2	2	28
	Neutrophil	2	14	338	30	19
	Neutrophil - activated	1	9	20	542	26

The network’s overall performance metrics also showed improvement, with precision reaching 87.5% ± 2.1% (up from 41.9% ± 2.8%), recall of 83.0% ± 2.0% (previously 41.2% ± 2.0%), and an F1 score of 82.0% ± 1.6% (compared to 40.9% ± 1.8%). Therefore, removing monocytes and activated monocytes reduced issues within the training data by focussing on cells with distinguishable morphological features during activation.

The confusion matrix in Table 4 shows 338 lymphocytes were correctly classified, with 108 misclassified as activated lymphocytes, and minimal confusion with neutrophil classes. Similarly, 440 activated lymphocytes were correctly identified, with only 57 misclassified as non-activated lymphocytes. Neutrophils showed positive classification with 338 correctly identified, and relatively minor misclassifications distributed mainly to activated neutrophils (30). The highest accuracy was observed for activated neutrophils, with 542 correctly classified and only 20 misclassified as non-activated neutrophils. The non-detection rates (Table 4) were notably low across all cell types, with 5 lymphocytes, 28 activated lymphocytes, 19 neutrophils, and 26 activated neutrophils not detected. As a useful network for discriminating between activated and non-activated lymphocytes and neutrophils was trained, a network was trained for monocyte activation.

Training a monocyte-specific model demonstrated improved performance compared to the previous models where monocytes were included alongside other cell types (Tables 5, 6). For non-activated monocytes, the model achieved a precision of 95.8% ± 4.1% (compared to just 5.2% ± 2.2% in the six-class model) and recall of 98.3% ± 3.5% (previously 14.1% ± 9.4%). This resulted in an F1 score of 97.0% ± 2.8%, representing an improvement from the previous 7.4% ± 3.3% (Table 5). Activated monocytes also showed enhancement in classification performance, with precision of 96.7% ± 7.5% (up from 6.67% ± 14.9% in the six-class model) and recall of 75.3% ± 22.2% (previously just 2.5% ± 5.6%). This produced an F1 score of 93.0% ± 16.6%, higher than the 3.6% ± 8.1% from Table 1. However, the relatively larger standard deviation in these metrics for activated monocytes suggests some variability across the five-fold cross-validation, potentially due to the smaller sample size of activated monocytes in the dataset or variations in activated morphology.

**TABLE 5:** Fold training outcome of stimulated monocytes and non-stimulated monocytes of a YOLOv4 network model. Detailed breakdown of the Precision, Recall and F1 score per blood cell subtype of the 5-fold cross validation of the binary network. Values are in percentage.

Cell type	Precision	Recall	F1 score
Monocytes	95.8 ± 4.1	98.3 ± 3.5	97.0 ± 2.8
Monocytes - activated	96.7 ± 7.5	75.3 ± 22.2	93.0 ± 16.6
Network	96.2 ± 4.5	86.8 ± 11.5	90.0 ± 9.7

**TABLE 6:** Fold training outcome of stimulated monocytes and non-stimulated monocytes of a YOLOv4 network model. Confusion matrix of one fold of the five-fold cross-validated model of the YOLOv4 model trained to discriminate between lymphocytes, activated lymphocytes, neutrophils, and activated neutrophils.

		Prediction		
		Monocyte	Monocyte - activated	Non detected
Ground truth	Monocyte	18	0	0
	Monocyte - activated	1	9	1

The confusion matrix in Table 6 reveals positive classification patterns. All 18 non-activated monocytes in the test set were correctly classified, with no false negatives or misclassifications. For activated monocytes, 9 out of 11 were correctly identified (81.8%), with 1 being misclassified as a non-activated monocyte (9.1%) and 1 not detected (9.1%). These results demonstrate the model’s strong capability to discriminate between the activation states of monocytes when trained specifically on this cell type.

The model trained to discriminate activated and non-activated leukocytes was used to test whether the white cell differential network could detect activated white cells in a simulated infection. For this, leukocytes were incubated with *E. coli* from a clinical isolate available in the lab. Based on the optical density at 600 nm, different dilutions of *E*. *coli* were prepared in media and left to incubate for 6 h.

### Growth dynamics of *E. coli* in samples

**FIGURE 4:**
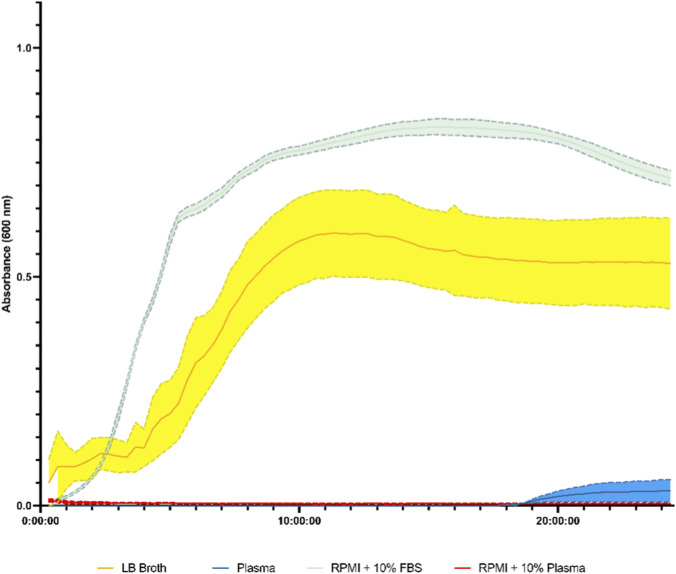
Growth curves of *E. coli* isolated from a clinical isolate from a urinary tract infection in different media. The media used were LB broth liquid media, fresh human plasma, fresh human plasma with 10% foetal bovine serum, RPMI with 10% foetal bovine serum, and RPMI with 10% fresh human plasma over 24 h. Absorbance measurements were taken every 20 min. The x-axis represents time in minutes, and the y-axis represents absorbance, which measures bacterial growth. n = 3 standard deviation as shaded areas.

Figure 4 displays the growth curves of *E. coli* from a clinical isolate under different growth conditions over a 24-h period. The black line represents the growth of *E. coli* in LB broth liquid media shows rapid growth. The blue curve depicting the growth of *E. coli* in human plasma shows a much slower growth rate compared to the LB curve, with lower maximum absorbance values. This suggests that the human plasma environment is less favourable for *E. coli* growth than the LB broth medium. The RPMI with 10% Plasma curves have no growth observed over 24 h. The RPMI with a 10% FBS curve displays better growth than LB Broth.

### Bacterial infection detection

**FIGURE 5:**
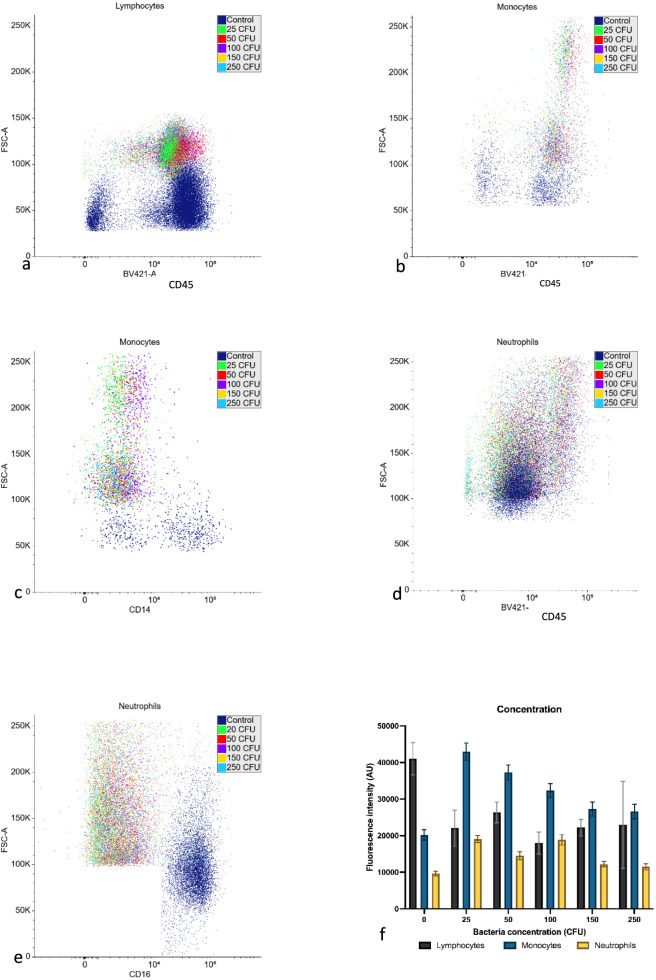
Flow cytometry verification of the effect of the incubation of the seeding concentration of *E. coli* within media with leukocytes (represented is one iteration of 3 donors × 3 replicates). **(a)** Flow cytometry scatterplot for lymphocytes after different seeding concentrations plotting CD45 probe fluorescence against forward scatter (FSC-A) in arbitrary units. **(b)** Flow cytometry scatterplot for monocytes (CD45/FCS-A). **(c)** Flow cytometry scatterplot for monocytes (CD14/FSC-A) in arbitrary units. **(d)** Flow cytometry scatterplot for neutrophils (CD45/FSC-A) **(e)** Flow cytometry scatterplot for neutrophils (CD16/FSC-A). **(f)** Bar chart comparing fluorescence intensity change against seeding concentration for lymphocytes, monocytes and neutrophils. The intensity was measured against the CD45 marker for lymphocytes, CD14 for monocytes and CD16 for neutrophils (n = 3, full statistical breakdown in Supplementary Figure SA1).

Flow cytometry was used to verify the activating effect of the bacterial seeding into the media (Figure 5). The figure illustrates the varying activation responses of lymphocytes and neutrophils to different bacteria concentrations, showing responses to the bacterial stimuli in the media in all samples tested. Looking at the scatter plots, the cell size increases across subtypes with an increase of fluorescence intensity for monocytes and neutrophils, suggesting an upregulation of CD14 and CD16 expression on the cell surface. Lymphocytes appear to decrease in fluorescence intensity for CD45 followed by a peak at 50 CFU/mL with a plateau after 100 CFU/mL.

**FIGURE 6:**
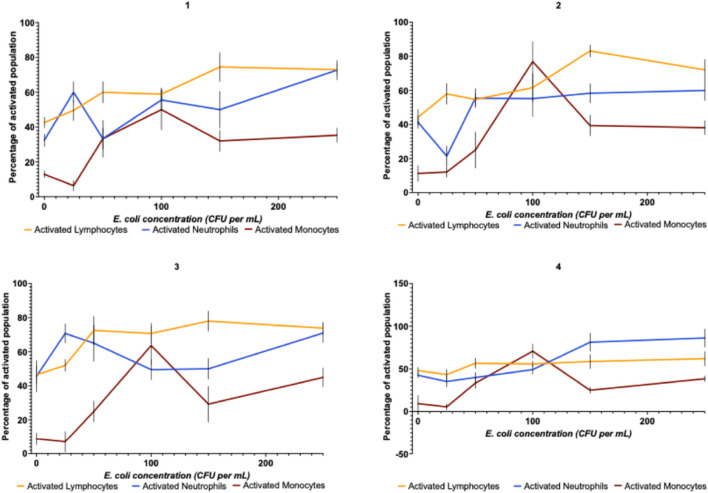
Total activated percentage of total cell populations for lymphocytes, neutrophils, and monocytes after detection and classification using trained YOLOv4 networks when incubating leukocytes with different concentrations of *E. coli* within the media. White cells were incubated for 6 h with different concentrations of colony-forming units of *E. coli*. The network trained on chemically stimulated white blood cells. Each graph represents different individual donors; each donor made three donations. Error bars represent differences between donor samples (4 donors × 3 replicates).

Figure 6 consists of four subplots, each representing different individual volunteers (1, 2, 3, and 4), each depicting the percentage of sub-population activated for three cell types, lymphocytes, neutrophils, and monocytes, against varying bacterial concentrations (CFU/ml; and a non-spiked control). The activation was defined as cells detected and classified as activated by trained YOLOv4 networks; alternative cell states such as apoptosis and necrosis could not be detected using this method. In subplot 1, the activation percentage for both the lymphocytes and the neutrophils shows a pattern, with the lymphocytes generally having a bigger activation percentage than the neutrophils. Both cell types reach peak activation around 100 CFU/mL bacterial concentration. Within the second subplot, there is a smoother activation curve for both cell types, with the lymphocytes peaking at around 100 CFU/mL and the neutrophils reaching maximum activation at a higher bacteria concentration of around 150 CFU/mL. In subplot 3, the activation patterns are similar to subplot 1, forming a pattern, with the lymphocytes having higher activation than the neutrophils across most bacteria concentrations. Both cell types reach their peak activation around 100 CFU/mL. For subplot 4, the graph displays a distinct pattern, where the neutrophil activation remains relatively low until around 150 CFU/mL, after which it sharply increases to a high level. Lymphocytic activation increased gradually, reaching a moderate level at higher bacteria concentrations. Overall, the peak of monocyte activation occurred using around 100 CFU/mL of bacteria. At higher CFUs, this decreases to around 30%–40% of total monocytes.

**FIGURE 7:**
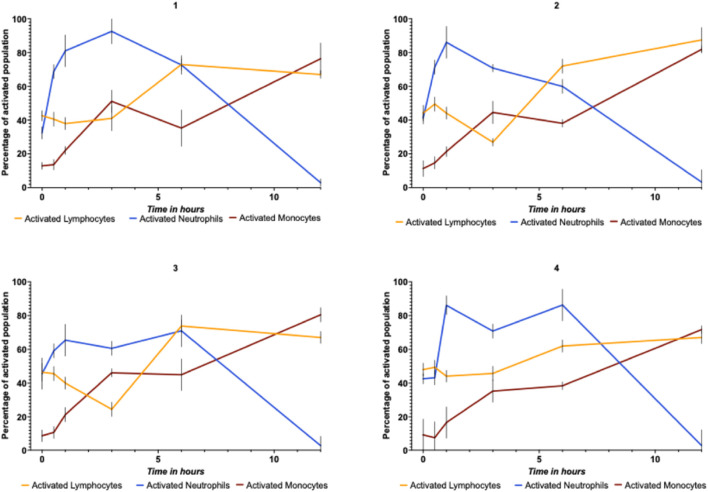
Inferred total activated percentage of total cell populations for lymphocytes, neutrophils, and monocytes after detection and classification using trained YOLOv4 networks when incubating leukocytes with 250 CFU of *E. coli* over 12 h of incubation using trained YOLOv4 networks. The detection was achieved using a trained neural network on chemically stimulated white cells but inferred on bacterial stimulated cells. Each graph represents three independent repeats from one donor. Each graph is from g a different donor. Error bars represent differences between donor samples (4 donors × 3 replicates).

Figure 7 displays four donors showing the percentage of activated lymphocytes and neutrophils over time with non-spiked controls. In subplot 1, neutrophil activation starts near 35%, rising to ∼85% at 180 min, while lymphocytes rise from 20% to 70% over 6 h. Subplot 2 shows lymphocytes beginning higher (∼40%) than neutrophils (∼25%) with both peaking around 100 CFU/mL. Subplot 3 peaks at 60 min rather than 180 min, and subplot 4 shows delayed neutrophil activation (∼150 min) relative to gradual lymphocyte increase. Subplot 2 exhibits a similar pattern, where neutrophil activation again begins higher than lymphocyte activation, then peaks at above 80% after one hour, followed by a gradual decline. Lymphocyte activation, on the other hand, starts low but increases gradually over time, surpassing the neutrophil activation after six hours of incubation with *E. coli*. In subplot 3, both lymphocytes and neutrophils show a similar activation pattern as the previous plots, with a peak around the middle time point (180 min for neutrophils) and lower activation at the beginning and end of the time course. Subplot 4 demonstrates a distinct activation profile, where the neutrophil activation starts high, drops to a minimum around 180 min, and then rises again towards the end. In contrast, lymphocytic activation remains relatively low initially, increases gradually, and surpasses neutrophil activation at the later time points. Activation of monocytes, inferred by the networks, showed an overall increasing trend of around 10%–20% beginning at 30 min of incubation, then steadily increasing to 70%–80% after 12 h. Overall, the four figures display similar activation kinetics for leukocytes over time, with different patterns observed across the four subplots, as detected by the white cell differential model after incubating white cells with live bacteria. However, the statistical analysis does not indicate significant differences in these temporal patterns.

**FIGURE 8:**
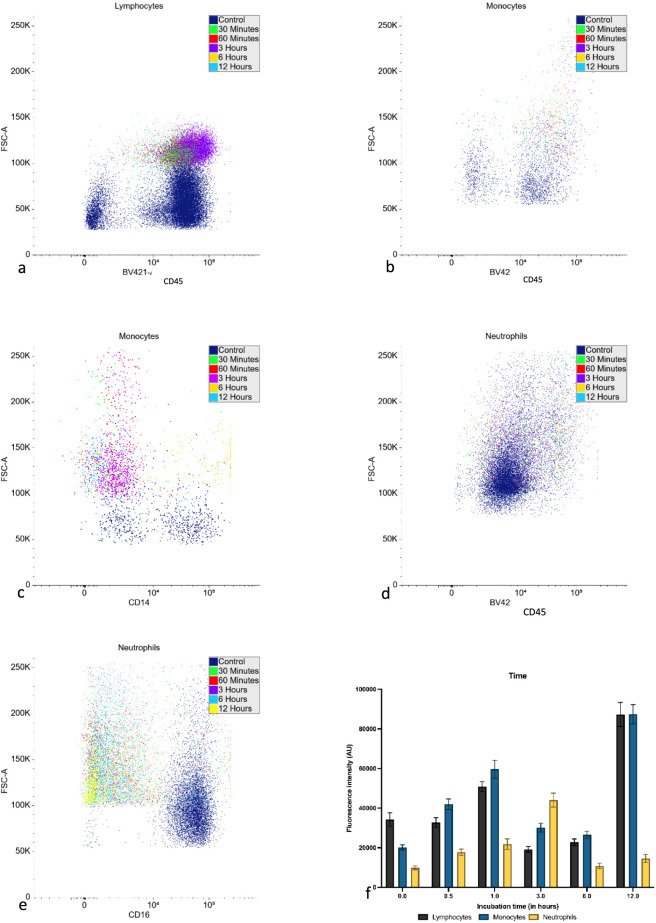
Flow cytometry verification of the effect of incubation time after seeding 250 CFU of *E. coli* within media with leukocytes (represented is one iteration of 3 donors × 3 replicates). **(a)** Flow cytometry scatterplot for lymphocytes after different incubation time plotting CD45 probe fluorescence against forward scatter (FSC-A) in arbitrary units. **(b)** Flow cytometry scatterplot for monocytes (CD45/FSC-A). **(c)** Flow cytometry scatterplot for monocytes (CD14/FSC-A) in arbitrary units. **(d)** Flow cytometry scatterplot for neutrophils (CD45/FSC-A). **(e)** Flow cytometry scatterplot for neutrophils (CD16/FSC-A) in arbitrary units. **(f)** Bar chart comparing fluorescence intensity change against incubation time for lymphocytes, monocytes and neutrophils. The intensity was measured against the CD45 marker for lymphocytes, CD14 for monocytes and CD16 for neutrophils (n = 3, error bars as standard deviation, full statistical breakdown available in Supplementary Figure SA2).

Flow cytometry shows different patterns of fluorescence between the control and treated samples (Figure 8). Suggesting that the presence of bacteria has altered the leukocyte characteristics to the extent the neural networks can detect the changes. Even after a half-hour incubation with bacteria, the leukocytes generally appear to have increased in cell size and fluorescence intensity (using specific CD markers–see Figure 8 for details). Fluorescence intensity gradually increases until before or at 3 h of incubation, after which the cell intensity continues to decrease gradually. Lymphocytes and monocyte fluorescence intensity peak at 12 h of incubation, suggesting the remaining cells have upregulated the expression of the specific CD markers.

### Testing activation inference in whole blood

**FIGURE 9:**
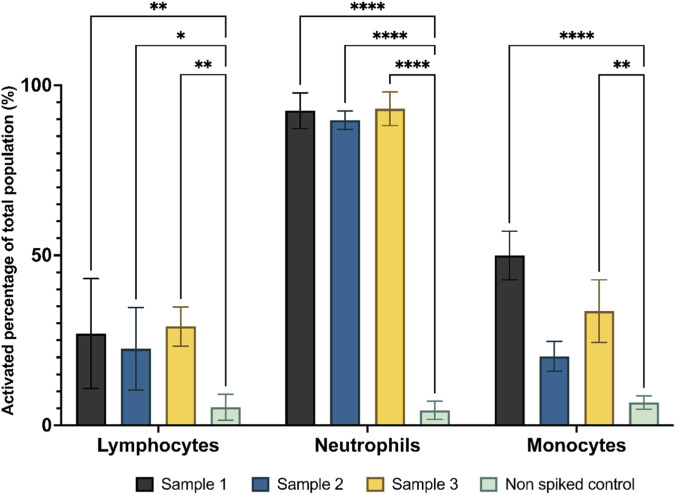
Detection of activated white blood cells from bacteria-spiked whole blood using a trained neural network. A trained YOLOv4 model was used to distinguish between activated and non-activated leukocytes. Each colour represents a different donor who has donated on three separate occasions (3 donors × 3 replicates). Bars represent the percentage of activated cells per cell type over total cell population count (activation percentage). Statistical significance between control and bacteria-seeded samples was determined using multiple t-tests with Holm-Šídák correction for multiple comparisons. Significance thresholds were set at p < 0.05 (), p < 0.01 (**), and p < 0.0001 (***).

Figure 9 presents the results of detecting activated white blood cells from spiked whole blood samples (11.11 ± 4.79 CFU per ml, verified by plate count) using a two-stage neural network approach. The network first identified blood cells within whole blood and isolated white blood cells. The second network then analysed the white cells, the white cell differential network, trained to distinguish between activated and non-activated leukocytes, which inferred the cell’s status. For lymphocytes, the percentage of activated cells ranged from approximately 15%–25% across the three donors. Sample 1 (black) exhibited the highest lymphocyte activation at around 25%, while Sample 2 (blue) showed the lowest activation at approximately 15%. In the case of neutrophils, a higher proportion of cells were detected as activated, ranging from around 90%. Sample 3 (yellow) showed the highest neutrophil activation, followed closely by Sample 1 (black). Sample 2 (blue) had the lowest neutrophil activation at around 90%. For monocytes, the results are a bit more varied. Monocyte activation was found to be between 25% and 50%, most likely due to the differences between individual differences in their respective immune systems.

## Discussion

The main goal of this paper was to develop a neural network model capable of detecting activated white blood cells in response to bacterial stimuli and to characterise its performance relative to Flow Cytometry. The activation of white cells was initially tested using known reagents. This was verified with flow cytometry. Levels of CD16 expression in neutrophils appeared low (Figure 2). For lymphocyte activation, PHA (15 μg/mL) and Con A (4 μg/mL) were chosen based on their well-documented ability to induce lymphocyte proliferation and activation (Ando et al., 2014; Simon-Molas et al., 2018). LPS at 0.1 μg/mL was selected as it represents a physiologically relevant concentration that effectively triggers inflammatory responses within monocytes without causing excessive cellular toxicity (Gomes et al., 2010). Similarly, the concentration of fMLP (43.8 μg/mL) used for neutrophil activation was based on dose-response studies demonstrating optimal neutrophil stimulation without inducing premature cell death or exhaustion (Vulcano et al., 1998). A standardised 16-h incubation period was applied across all leukocyte populations to maintain experimental consistency, as this timeframe has been widely used in published studies for lymphocyte and monocyte activation with PHA, Con A, and LPS (Gomes et al., 2010; Ando et al., 2014; Simon-Molas et al., 2018). However, this extended duration likely had differential effects on the various cell types, particularly neutrophils. The low CD16 expression observed in neutrophils could be attributed to the length of the experiment, whereby the cells could have activated and then died from exhaustion. fMLP activation of neutrophils is relatively fast in comparison to other leukocyte subtypes, taking only a few minutes (Rochon and Frojmovic, 1993). Thus, it is entirely plausible the cells have either downregulated the CD16 marker after prolonged activation or succumbed to cell death from exhaustion (Pérez-Figueroa et al., 2021). While a shorter incubation period might have been optimal for neutrophils, the standardised protocol was necessary to generate a consistent training dataset for the neural network across all cell types. While the CD45 marker was primarily used here to distinguish lymphocytes from monocytes and neutrophils with their respective co-expression of CD45 and CD14/16; looking at the CD45RA/RO, isoforms of the CD45 protein distinguishing between naïve (RA) and memory (RO) T lymphocytes, co-expression would be a better measure of lymphocyte activation than using fluorescence intensity change and cell size change alone (Clement, 1992). Discriminating between isoforms, along with a shorter incubation time for neutrophils, could be something to investigate for future studies on the subject and/or testing the feasibility of a network being able to distinguish one expression form from another.

The YOLOv4 white cell differential model demonstrated varying capabilities in discriminating between stimulated and unstimulated leukocytes. As shown in Table 1, the model achieved moderate performance for lymphocytes and neutrophils with F1 scores around 56%–58% but struggled significantly with monocyte classification, yielding remarkably poor F1 scores of just 7.4% and 3.6% for non-activated and activated monocytes, respectively. This performance disparity likely comes from subtle morphological changes in activated monocytes that were challenging for the multi-class model to detect, compounded by the evident class imbalance in the training data, with notably fewer examples of monocytes overall, as confirmed by the confusion matrix (Table 2).

When all monocytes were removed from the training data, the network’s performance improved noticeably across all remaining cell types (Tables 3, 4), with F1 scores for lymphocytes and neutrophils increasing to 80%–91%. This improvement suggests that the inclusion of poorly differentiated cell types adversely affected the overall network performance. Most significantly, when a dedicated model was trained exclusively for monocyte activation status (Tables 5, 6), the performance metrics improved remarkably to F1 scores of 97.0% and 93.0% for non-activated and activated monocytes, respectively.

This aligns with the biological understanding of leukocyte activation morphology. The activation of circulating lymphocytes and neutrophils does affect their overall morphology (Lin et al., 2015; Sergunova et al., 2023). For example, T-lymphocytes change their morphology when activated, whereby flattening and either elongating or keeping a wider but round shape. More generally, when presented with antigens, lymphocytes undergo activation and proliferation as a sign of the cellular adjustments, which are required to build a successful immune response. The decision to employ transfer learning, using a pretrained YOLOv4 backbone rather than training the network entirely from scratch, was driven by both data efficiency and performance considerations. Transfer learning enables the reuse of low-level visual features such as edges, gradients, and textural cues that are broadly transferable across imaging contexts, including brightfield microscopy. This initialisation accelerates convergence and stabilizes training, as the model begins from a well-structured feature space rather than random weights. Importantly, it also mitigates overfitting when working with limited cell images per class, a common constraint in biomedical datasets. In our preliminary experiments, models trained from random initialization exhibited faster overfitting and reduced validation F1-scores compared with the pretrained initialization, supporting the rationale for adopting a transfer-learning approach in this study.

When not activated, lymphocytes are typically small in size (8–10 µm in diameter) with a high nucleus-to-cytoplasm ratio and densely packed chromatin (Cano and Lopera, 2013). This changes when these quiescent cells respond to an antigenic stimulation. Lymphocytes dramatically change into larger, actively proliferating cells known as lymphoblasts. The cytoplasmic expansion observed in lymphoblasts explains the increase in cell size, which is a consequence of the elevated RNA content necessary to meet the heightened demands for protein synthesis in these actively proliferating cells. Additionally, the previously condensed chromatin is now loosened to facilitate gene expression and replication (Bediaga et al., 2021). Parallel with the decondensation of chromatin, the nucleoli within the nucleus exhibit an increase in size and shape. This morphological feature reflects the upregulated production of ribosomal RNA to meet the increased translational requirements of the activated lymphocyte (Sadeghi Shoreh Deli et al., 2022).

As part of the innate immune system, neutrophils, part of the first line of defence against infection, are able to rapidly adapt morphological features to an antigenic stimulus. When in their quiescent state, neutrophils have a multi-lobed, segmented nucleus and a cytoplasm accompanied by multiple structures containing various antibacterial compounds such as oxidants, proteinases and cationic peptides (Moraes et al., 2006; Sergunova et al., 2023). When activated, the granules within the neutrophils are released into the extracellular environment to cause damage to the pathogen. Because of this, the intracellular morphology of neutrophils becomes less discernible. Neutrophil activation is accompanied by increased motility and chemotactic responsiveness, facilitated by dynamic cytoskeletal rearrangements that enable efficient migration towards the site of infection (Morikis and Simon, 2018). Notably, neutrophils, when attacking a foreign body such as a bacterium, will produce an extracellular net or trap. However, none were observed here. To prepare the trap, the cells undergo a few changes from rearranging their nucleus, spreading, membrane disruption and even cell disintegration (Sergunova et al., 2023). Most of these are within the order of magnitude to be observed under the microscope. Altogether, these morphological changes are detected by the white cell differential network and used to discriminate between the normally circulating cells and their activated counterparts, as evidenced by the confusion matrix. While eosinophils and basophils were not explicitly included, their rarity in peripheral blood and morphological resemblance to neutrophils means that any misclassification would have minimal impact on network performance. Incorporating these subtypes in future training could further improve generalisability.

The significantly different performance between the multi-class (Tables 1, 2) and monocyte-specific (Tables 5, 6) models suggests that while monocytes undergo activation-related morphological changes, these alterations may be more subtle or qualitatively different from those observed in lymphocytes and neutrophils. While the multi-class model struggled with monocyte classification (F1 scores of only 7.4% and 3.6%), the specialised model achieved remarkable accuracy (F1 scores of 97.0% and 93.0%).

Typically, monocytes exhibit a characteristic large (12–20 µm in diameter) reniform nucleus and a moderate amount of finely granulated cytoplasm (Espinoza and Emmady, 2024). Upon activation, their morphological changes appear more constrained when compared to the dramatic transformations observed in other leukocytes. In suspension, activated monocytes largely maintain their spherical shape but exhibit subtle alterations in nuclear presentation, transitioning from a single-lobed to a multi-lobed nucleus - changes that correlate directly with shifts in phenotypical expression as activation progresses toward differentiation into macrophages or dendritic cells, influenced by cytokines like CSF-1, CSF-2, and IL-34 (Menzyanova et al., 2019; Chaintreuil et al., 2023). During this differentiation process, more pronounced changes emerge, including significant cytoplasmic expansion with vacuolisation to accommodate phagocytic activity (Sieweke and Allen, 2013). These relatively subtle initial morphological alterations may explain why the multi-class model failed while the dedicated classifier succeeded. Additionally, monocyte isolation yielded fewer images, and despite oversampling, morphological subtlety and isolation-induced variability likely contributed to limited model performance.

The initial trained white cell differential model’s inability to discriminate between unstimulated and activated monocytes could be due to the material choice of the microfluidic chip, (Tables 1, 2). Topaz is a low-binding, low-absorbent material. And so, while other cells may easily express their morphological change in circulation, monocytes may require a surface to fully express their morphological change, which can then, in theory, be seen by the microscope, while the changes the monocytes express in circulation cannot be detected (changing their nuclei formation). However, as the cells were in flow when captured, it is unlikely that monocytes would have had the opportunity to attach to the channel surface. Another potential issue could be depletion during culture. Additionally, the need for more examples of the white cell differential network to train on. Due to the low number of circulating monocytes, isolating them from whole blood and imaging has proven challenging without drawing large volumes of blood (Boyette et al., 2017), which, itself, can pose issues. The trick here may be utilising the number of circulating monocytes to distinguish the presence of infection itself (Patel et al., 2017). However, this could still cause an issue where the monocyte training data is poisoned by the other cells (Shorten and Khoshgoftaar, 2019).

The relatively low frequency of monocytes within a blood sample compared to other leukocytes may contribute to the inability of the leukocyte activation network from Tables 1, 2 to discriminate between activated and non-activated monocytes. Monocytes typically constitute only 5%–8% of circulating leukocytes in peripheral blood (Hoffbrand et al., 2006). This low abundance poses a significant challenge for obtaining sufficient training examples for the neural network. To collect an adequate dataset of monocyte images for robust neural network training would require processing substantially larger blood volumes than the standard 5 mL typically collected for research purposes as noted in your methodology. While concentration techniques such as density gradient centrifugation followed by adhesion-based isolation were employed in this study, these methods yield relatively lower counts of monocytes compared to other cell types, with the isolation procedure itself potentially altering cell morphology and activation states (Fluks, 1981; Menck et al., 2014; Nielsen et al., 2020). Therefore, the monocyte-specific model was not reintegrated into the primary four-class pipeline to avoid error propagation from the initial cell-type classifier. Another potential issue could come from imperceptible background features within the images of other cell subtypes within the training dataset, rendering it incapable of discriminating the monocytes (Oakden-Rayner et al., 2020; Shan et al., 2024). The reference to ‘background features’ refers to subtle contextual cues (e.g., debris or nearby platelets) that may correlate spuriously with certain classes if not evenly represented. These artefacts likely affected the multi-class model but were minimized in the monocyte-only dataset. Taking these into account and considering the ability of the monocyte-only network to accurately distinguish between activated and non-activated monocytes (Tables 5, 6), the microenvironment in which the cells are observed does not seem to affect the training of the monocyte network therefore, it could be ruled out as the likely cause for the problem. The frequency of monocytes within the dataset could also be one likely factor. Increasing the number of monocyte examples within the dataset would not only increase neural network accuracy but also narrow down the cause of the issue.

Despite this limitation, the trained white cell differential model proved effective in detecting activated lymphocytes and neutrophils when incubated with live *E. coli* bacteria (Figures 6, 7). The heterogeneity in activation profiles observed across the different subplots in Figures 6, 7 reflects biological variability that may stem from multiple factors. The data suggests temporal patterns in leukocyte activation, with neutrophils exhibiting an apparent rapid response curve that peaks between 60 and 180 min post-stimulation before declining. Meanwhile, lymphocyte activation demonstrates a more gradual increase with elevation at later time points. However, it is worth noting that while these apparent patterns emerge visually in the data, statistical analysis revealed no significant differences between these activation profiles.

Leukocyte responses were verified by flow cytometry (Figures 5, 8). In some cases, two populations were observed in the neutrophil subset, or the total number of events decreased below that anticipated compared to the control. This would suggest either a heterogenous population from exposure to the pathogen or the cells dying from the extended stimulatory response, effectively dying of exhaustion (Nedeva, 2021). These findings align with the known roles of neutrophils as rapid responders in innate immunity and lymphocytes in adaptive immunity, which involves a more prolonged response.

Indeed, during the immune response, neutrophils would arrive first at the site of infection, where they fight pathogens by releasing other cytotoxic chemicals and reactive oxygen species, which would cause the recruitment of monocytes to the site (Chaplin, 2010). A curve for monocyte activation could not be calculated due to low numbers and the white cell differential network’s ability to discriminate activated from non-activated monocytes. The monocyte activation curve would be seen following neutrophil increase, albeit with a slight delay. Then, the macrophages, which arise from monocyte differentiation, begin phagocytosing infective agents, presenting antigens, and coordinating a longer-lasting immune response by stimulating lymphocytes (Chaplin, 2010). Typically, it would take many days for antigen-specific cells to clonally expand to mount an effective adaptive immune response.

While donor-level experiments were qualitatively consistent with flow cytometry trends, a direct quantitative correlation was not calculated. The present validation was limited to parallel visual and temporal comparison between methods. The observed lymphocyte activation pattern, while interesting, must be interpreted carefully. The timeframe of the experiments (12 h) is generally insufficient for a full primary adaptive immune response, which typically requires several days for significant clonal expansion and effector function development (Hoffbrand and Lewis, 1989; Hoffbrand et al., 2006). The detected lymphocyte activation may represent early activation events or innate-like responses from specific lymphocyte subsets rather than evidence of immunological memory. This accelerated activation likely reflects artefacts of the *in vitro* environment rather than physiological responses. Isolated lymphocytes in culture can demonstrate non-specific activation due to multiple factors, including mechanical stress during isolation, exposure to foreign serum components in the culture media, or altered cellular densities and spatial arrangements that differ significantly from *in vivo* conditions (Yassouf et al., 2022). Additionally, the absence of regulatory mechanisms in the complete immune microenvironment may permit activation processes that would otherwise be controlled in the body (Goldmann et al., 2024). Therefore, while the current system successfully detects cellular activation states, the temporal dynamics observed, particularly for lymphocytes, should not be directly extrapolated to *in vivo* immune responses without further validation in more physiologically relevant models. Future studies incorporating specific memory markers and longer observation periods would be needed to distinguish between primary and memory responses. Regardless, these observations highlight the complementary roles of the innate response (represented by neutrophils) and the developing adaptive response (represented by lymphocytes) in antimicrobial defence (Chaplin, 2010).

Cell morphology changes as cells age, with leukocytes demonstrating altered nuclear shapes, reduced membrane integrity, and decreased cytoplasmic granularity (Belhadj et al., 2023). These age-related changes could potentially influence sample classification accuracy, introducing an additional variable that warrants consideration in future validation studies. Fixation of leukocytes presents an alternative approach that could address some of the challenges encountered with live cell analysis. Chemical fixatives such as paraformaldehyde or glutaraldehyde preserve cellular morphology by cross-linking proteins, potentially capturing activation-specific structural features while preventing further morphological changes during processing and analysis (Qin et al., 2021). This approach offers several advantages, including extended sample stability, reduced biohazard risks, and standardisation of morphological features at specific time points post-activation. However, fixed cells often exhibit shrinkage, increased membrane rigidity, nuclear condensation and altered granule appearance compared to their living counterparts, which could impact the features used by neural networks for classification (Crawford and Barer, 1951; Chen et al., 2012). Future studies could explore whether a fixed-cell approach might improve detection consistency, particularly for monocytes, by comparing classification accuracy between fixed and unfixed samples across various time points post-activation.

The leukocytes were able to activate at all concentrations of bacteria down to around 25 CFU per ml in the medium (verified by plate count, 26.3 ± 2.34), which is about 2.5–25 times above the detection limit of other technologies, such as blood cultures for bloodstream infections (infections typically detect down to 1–10 CFU per mL; Opota et al., 2015). However, the accuracy of the count varied, with counts varying from 19 to 26 CFU per ml.

However, a follow-up experiment, seeding bacteria into a whole blood sample, showed the white cell differential network was able to identify activated white cells from a 30-min incubation Figure 9. Checking the seeding concentration of whole blood revealed a CFU per mL of 12, a concentration close to the detection limit of 1–10 CFU in clinical blood cultures. Therefore, it is not a stretch to envisage the possibility of the white cell differential network detecting the immune response to bacterial infection within this range, if not below. Researchers have demonstrated the effect of low concentrations of LPS (100 ng/mL) extracted from *E. coli* bacteria and its efficiency in causing the production of immune-related cytokines (Chen et al., 2017).

We tested the whole system on whole blood seeded with around 10 CFU/mL of *E. coli* (11.11 ± 4.79 CFU per ml), using two networks–the whole blood differential network trained on whole blood and the white cell differential network. The system isolated the white cell images from the whole blood in a preliminary step that excluded red cells and platelets. During operational inference, this process is fully automated: the whole blood differential network identifies leukocytes, which are automatically passed to the white cell differential network for activation classification without manual review. Manual curation of leukocyte images was performed only during initial training dataset preparation to ensure high-quality labels for training the white cell differential network. Then, the white cell differential neural network was loaded and performed the differential inference, discriminating between activated and non-activated white cells. Here, any false positive detections from the whole blood differential neural network were ignored as the white cell differential network was trained only on white cell data, thus reducing false detections. The whole inference took less than five minutes once the data was captured. Interestingly, in Figure 9, there was a strong activation of neutrophils due to the presence of bacteria within the spiked blood sample, in comparison to the lower percentage of activation of monocytes and lymphocytes. The low presence of activated lymphocytes can be explained by the short incubation time with bacteria. Neutrophils and monocytes, part of the innate immune system, respond to an *in vivo* bacterial presence to initial infection. Additionally, neutrophils are known to activate in response to stimulation with LPS (Gomes et al., 2010). Thus, it is possible to theorise that within such an infection, the neural network would be able to infer the presence of activated white cells.

The growth curves of *E. coli* in LB broth and human plasma (Figure 5) provide valuable context for interpreting the white blood cell activation patterns. The rapid growth observed in LB broth suggests a highly favourable environment for bacterial proliferation. In contrast, the slower growth in human plasma highlights the intrinsic antimicrobial properties of this environment, which likely contributed to the observed white blood cell activation profiles (Pont et al., 2020). Additionally, the limited or absent growth in human plasma indicates potential nutritional constraints that may restrict bacterial survival. Plasma may be deficient in vital metabolic substrates needed for bacterial replication because it lacks the complete nutritional profile of specialised bacterial growth media (Bochkov et al., 2016). This nutritional deficiency in plasma could significantly impede bacterial growth, explaining the lower growth curves for plasma.

Overall, this study demonstrates the potential of AI-based approaches for analysing cellular morphological responses to stimuli. The ability to accurately detect and quantify activated white blood cell populations could have significant implications for disease diagnosis, monitoring, and treatment. This could be applied to any pathology with a morphological manifestation, such as anaemias, where the morphological change is specific to the anaemia type and cause (Chaparro and Suchdev, 2019; American Society of Hematology, 2024). However, leukocyte changes are not uniquely diagnostic of bacterial infections, as morphological alterations can result from diverse conditions, including viral infections, inflammatory processes, autoimmune disorders, and systemic stress responses (Thieblemont et al., 2016; Chmielewski and Strzelec, 2018; Pozdnyakova et al., 2020; Sharma et al., 2023). The current diagnostic system should not be used in isolation; accompanying clinical assessment and patient history should be used in combination to ensure accurate and nuanced medical interpretation.

A critical consideration for this technology is the potential impact of false negatives in clinical settings. Failure to detect activated leukocytes could delay crucial treatment interventions, particularly in severe infections where timely diagnosis is essential (Joo et al., 2014). The prototype’s differential performance across cell types, robust for neutrophils but limited for monocytes, could create diagnostic blind spots for infections where monocyte activation predominates. The temporal dynamics observed, while non-significant, also suggest detection sensitivity may vary depending on the infection stage, potentially missing late-presenting cases where neutrophil activation has already declined.

These limitations necessitate careful clinical validation before implementation. Future work should quantify false negative rates at clinically relevant bacterial concentrations and establish concordance with gold standard methods. While offering advantages in speed and minimal sample handling, the consequences of missed activation signals require a measured approach to clinical integration. While technologies like deep-UV microscopy have demonstrated label-free haematological diagnostics, our system differs by using entirely off-the-shelf, low-cost optical and microfluidic components (Gorti and Robles, 2023; Gorti et al., 2023). The setup requires no specialised alignment or custom hardware and can be assembled with off the shelf components. Moreover, both techniques require minimal sample preparation, however deep-UV provides more granular data such as haemoglobin mass compared to total cell counts (Robles and Ojaghi, 2020).

Furthermore, as the technology can detect the activation of leukocytes, detecting infections from viruses and fungi by proxy could be possible. Additionally, this approach could be used to expedite leukaemia diagnosis by concurrently doing cell counts and morphological analysis. By analysing different cell types and spotting any anomalies, the dedicated neural network can offer a thorough blood count by analysing microscopy images. Then, using a follow-up network, assessing the morphology of leukocytes and detecting any alterations, including the presence of blast cells or other abnormal cells, would be able to indicate the presence of leukaemia (Döhner et al., 2022). This dual capability would enable healthcare diagnostic laboratories to detect and treat leukaemia more effectively by accelerating the diagnostic process, reducing human error, and delivering holistic results. Currently, to confirm a leukaemia diagnosis, both a differential cell count and blood smear are required; this technology could also be applied within veterinary medicine where only small amounts of blood are available for a diagnosis, especially for pathologies such as haemolytic anaemias in canines and felines (Swann et al., 2016; Maldonado-Moreno et al., 2023; Baldwin et al., 2024). It is worth considering the possibility of adding a red cell differential network to investigate red cell morphology anomalies, which are telltale of pathologies. One such example is sickle cell disease with crescent moon shape disease (Elendu et al., 2023). Future work could focus on improving the white cell differential model’s performance in distinguishing activated monocytes and exploring its applicability to other bacterial or viral pathogens. Another potential future work could be the initial discrimination of the leukocytes followed by a binary classification network responsible for the inference of the status of the white cells and or detection of the potential leukaemic status of cells.

## Conclusion

We demonstrated the successful development of white cell differential networks to detect and quantify activated leukocytes in response to bacterial stimuli within the whole blood environment. The system effectively detected activated white cells at low bacterial concentrations, showcasing the potential for expediting disease diagnosis and monitoring immune responses. Rivalling flow cytometry by its ease of use and potential integration of additional follow-up tests without the need for further sample handling or trained technicians. Future work should address the monocyte classification challenge; one main avenue should be to increase the imaging monocyte dataset. The integration of artificial neural networks, microfluidics, and microscopy promises to revolutionise haematological diagnostics, enabling more precise and accessible multi-pathology diagnostics.

## Data Availability

The raw data supporting the conclusions of this article will be made available by the authors, without undue reservation.
