# Supplementary material for: Stain-free artificial intelligence-assisted light microscopy for the identification of leukocyte morphology change in presence of bacteria
Source: Front Bioinform. 2026 Jan 6;5:1725145. doi: 10.3389/fbinf.2025.1725145 (PMC12816271; doi:10.3389/fbinf.2025.1725145)

Appendix: Stain-free artificial intelligence-assisted light microscopy for the identification of leukocyte morphology change in presence of bacteria.

Alexander Hunt[1], Holger Schulze[1], Kay Samuel[2], Robert B. Fisher[3], Till T. Bachmann[1]

1. Centre for Inflammation Research, Institute for Regeneration and Repair, The University of Edinburgh
2. Tissues, Cells & Advanced Therapeutics, Scottish National Blood Transfusion Service, NHS National Services Scotland, Jack Copland Centre, 52 Research Avenue North
3. School of Informatics, The University of Edinburgh

Correspondence author: Prof. Till Bachmann, till.bachmann@ed.ac.uk
Keywords: Artificial Neural Network, morphological analysis, YOLO v4, blood analysis

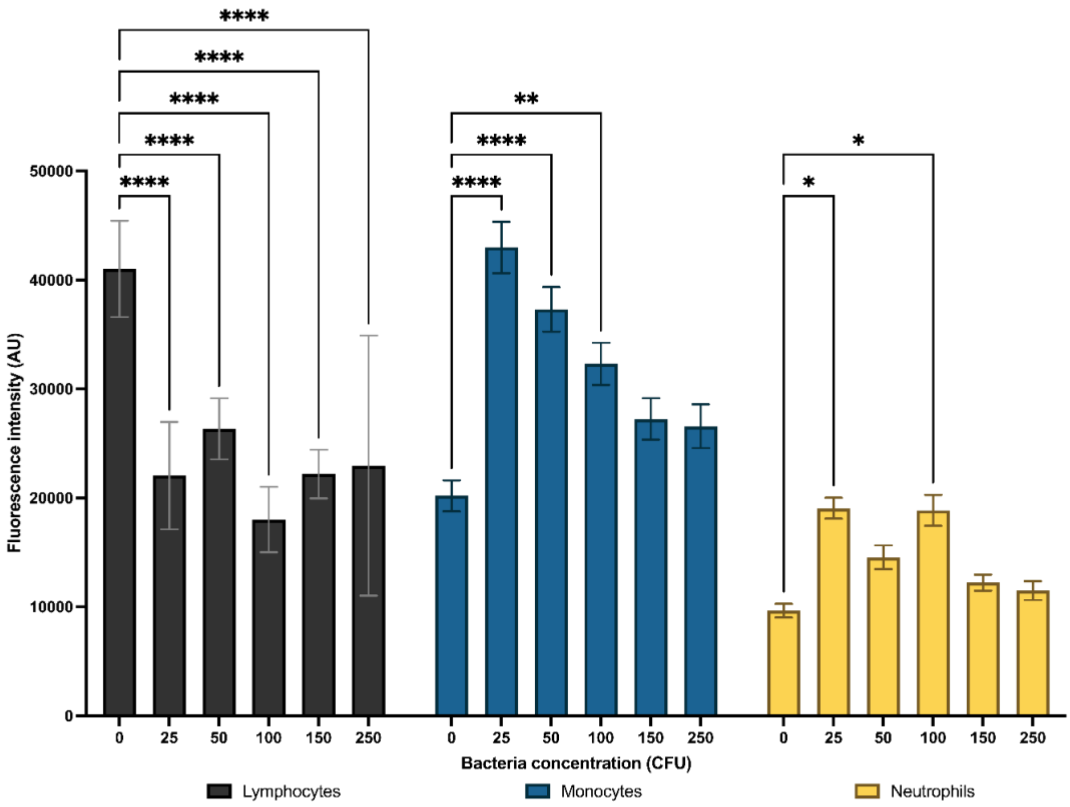

*Appendix figure 1: Fluorescence intensity of immune cells in response to different bacterial concentrations. The graph displays the mean fluorescence intensity (AU) with error bars representing the standard deviation for lymphocytes (black), monocytes (blue), and neutrophils (yellow) exposed to increasing bacterial concentrations (0, 25, 50, 100, 150, and 250 CFU, n=3). Asterisks indicate statistical significance: * $p < 0.05$, ** $p < 0.01$, **** $p < 0.0001$. Lymphocytes show the highest fluorescence at baseline (0 CFU) with significant decreases at all bacterial concentrations. Monocytes display peak fluorescence at 25 CFU with significant decreases at higher concentrations. Neutrophils show significantly higher fluorescence at 25 and 50 CFU compared to baseline, with a gradual decrease at higher bacterial concentrations.*

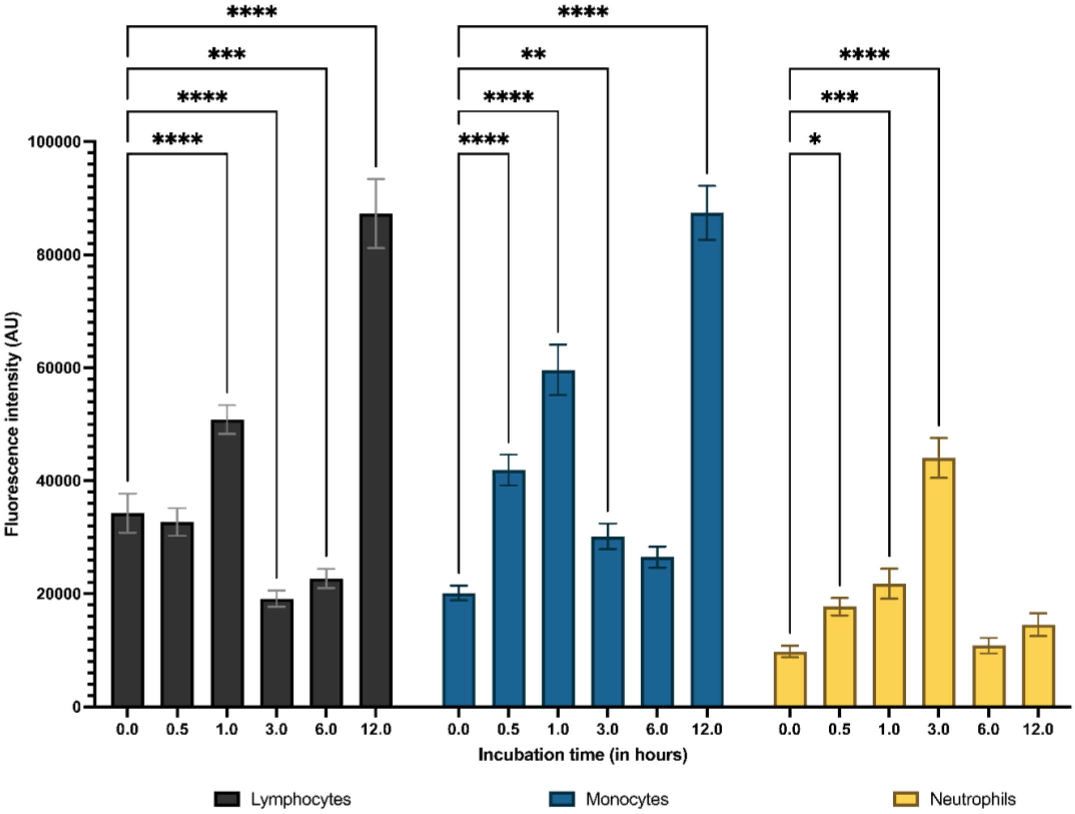

*Appendix figure 2: Fluorescence intensity of immune cells measured at various incubation time points. The graph displays mean fluorescence intensity (AU) with error bars representing standard deviation for lymphocytes (black), monocytes (blue), and neutrophils (yellow) measured at 0, 0.5, 1.0, 3.0, 6.0, and 12.0 hours of incubation (n=3). Asterisks indicate statistical significance: * $p < 0.05$, ** $p < 0.01$, *** $p < 0.001$, **** $p < 0.0001$. All three cell types show distinctive temporal patterns: lymphocytes exhibit peak fluorescence at 12.0 hours with significant differences from all earlier time points; monocytes demonstrate biphasic behaviour with peaks at 3.0 and 12.0 hours; neutrophils show a progressive increase, reaching maximum intensity at 3.0 hours followed by a decline. Statistically significant differences are observed between specific time points, as indicated by the connecting brackets.*

Flow Cytometry Gating Strategy

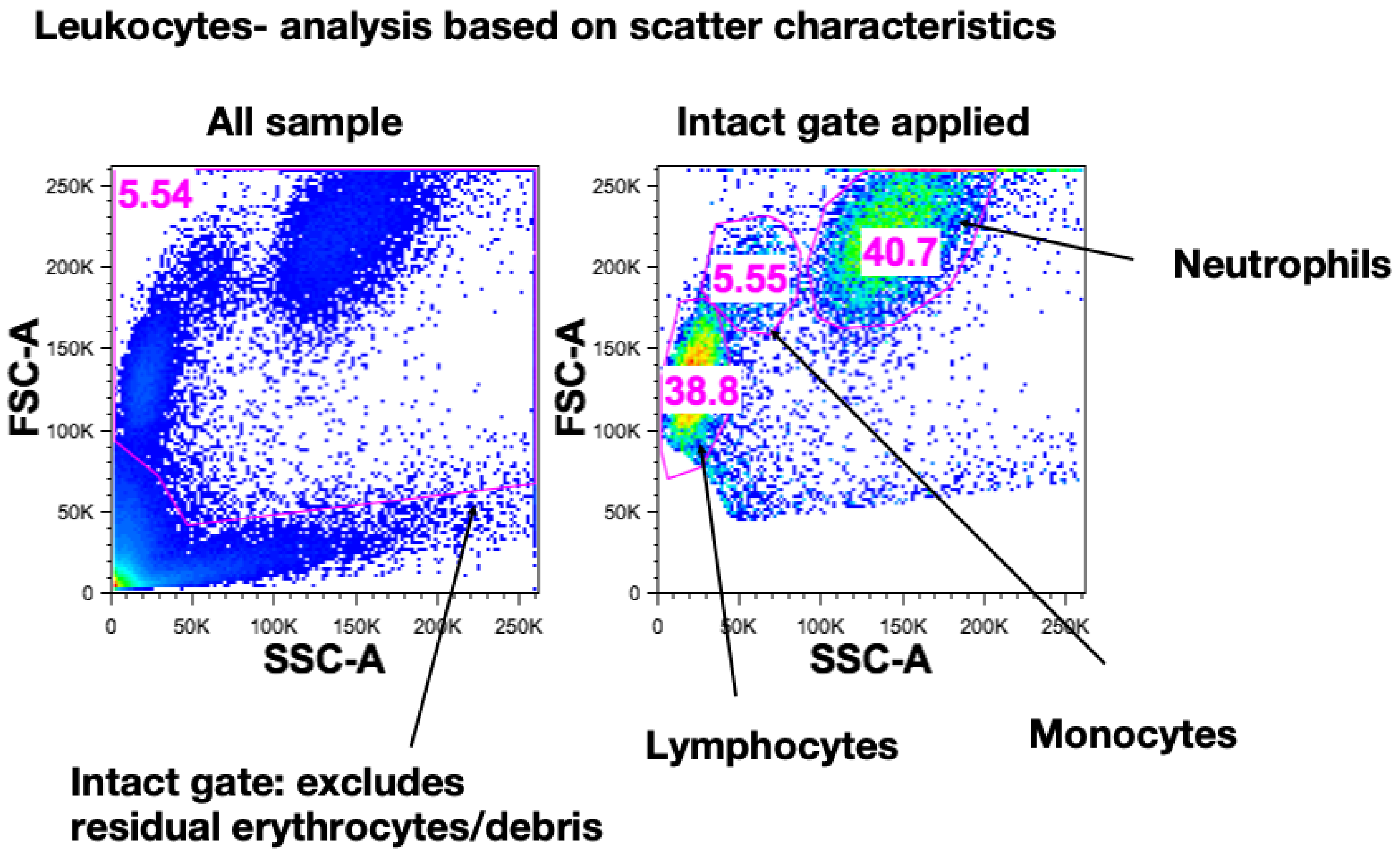

Lymphocyte-analysis based on scatter gating and ab staining

Lymphocytes CD45+CD14-CD16-

Intact gate applied

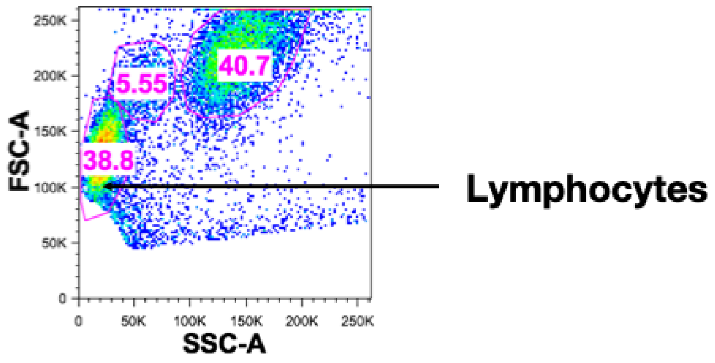

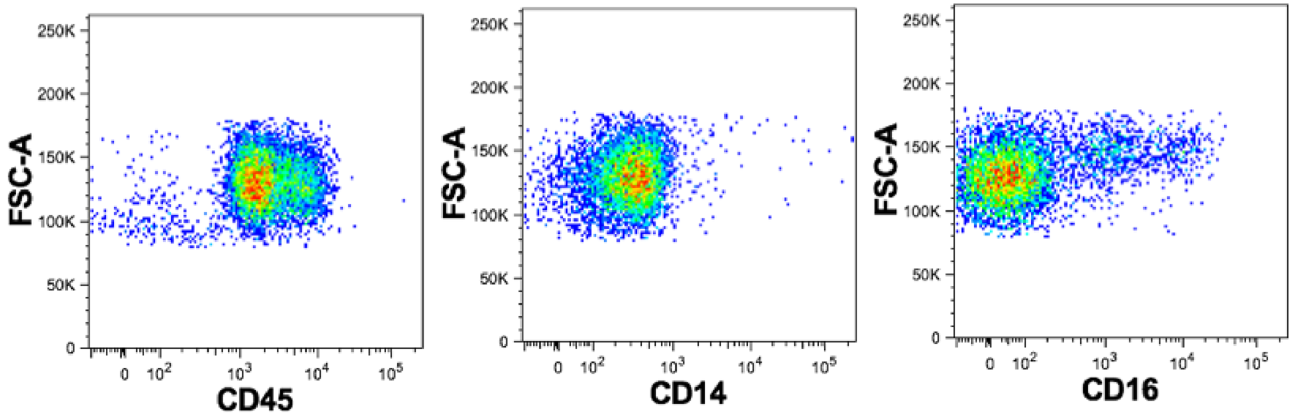

lymphocytes CD45+ **lymphocytes CD14-** **lymphocytes CD16-**

Monocyte-analysis based on scatter gating and ab staining

Monocytes CD45+CD14+CD16-

Intact gate applied

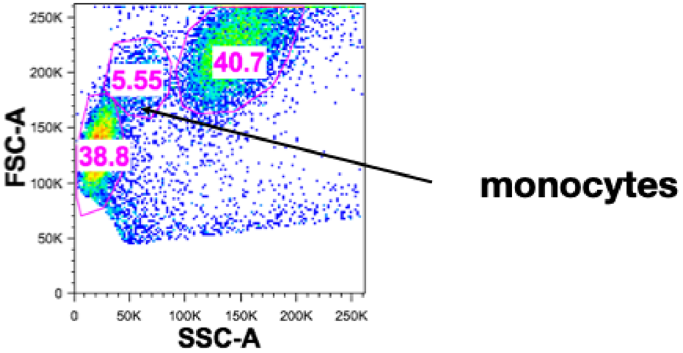

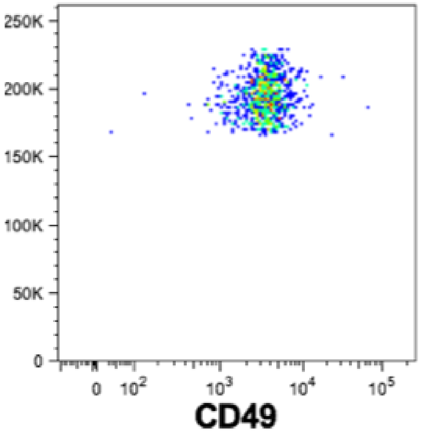

Monocytes CD45+

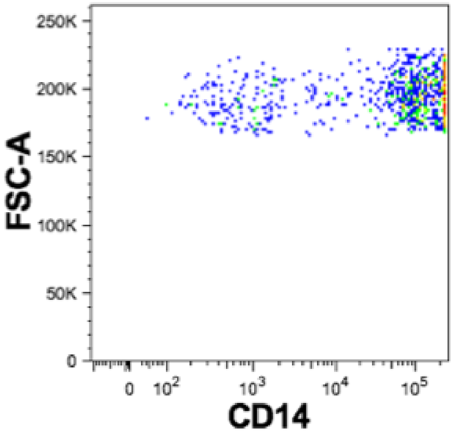

Monocytes CD14+

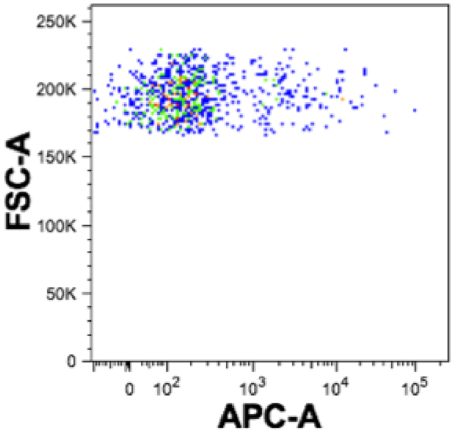

Monocytes CD16-

Neutrophil-analysis based on scatter gating and ab staining

Neutrophils CD45+CD14+CD16+

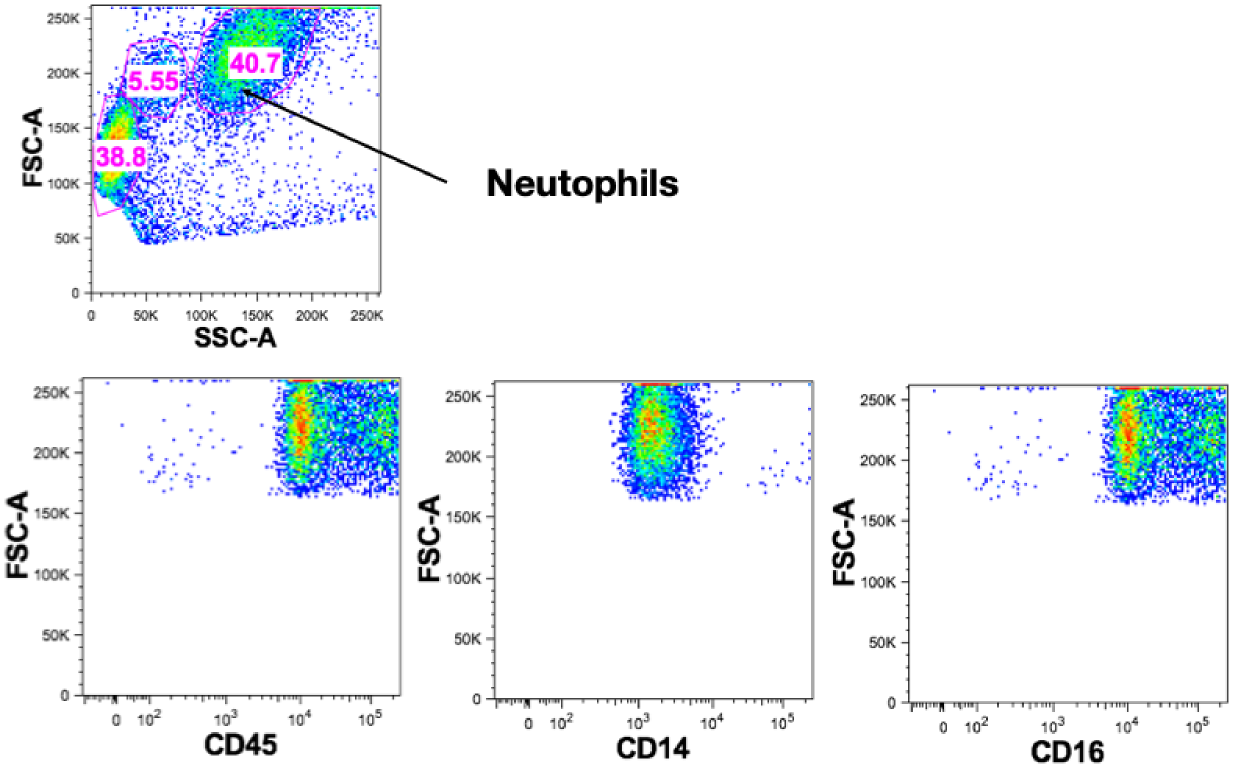

Neutrophils CD45+ **Neutrophils CD14+** **Neutrophils CD16+**

Leukocytes- analysis based on intact gate and ab staining

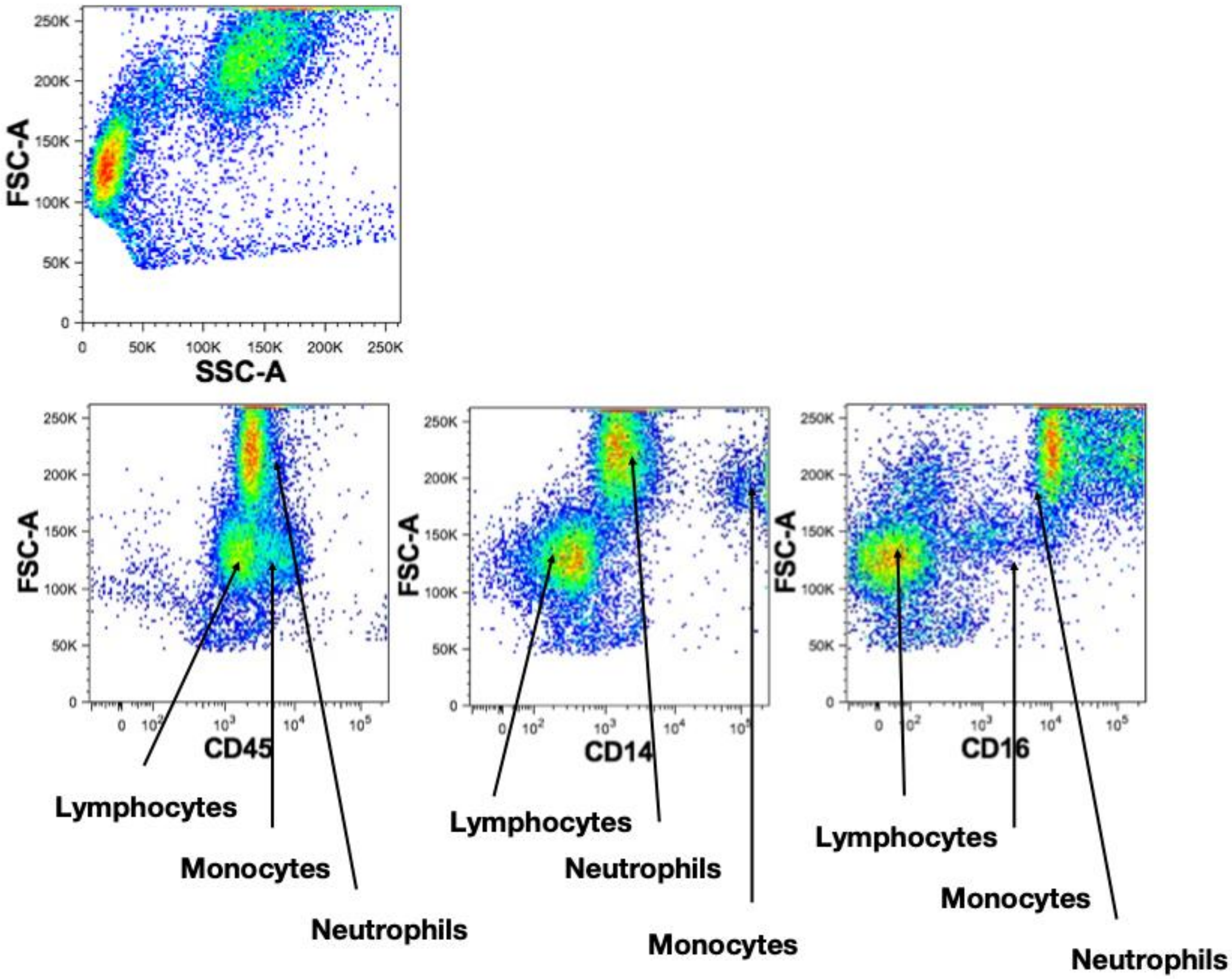

Erythrocytes and platelets analysis based on scatter characteristics

All sample | **Intact gate applied**

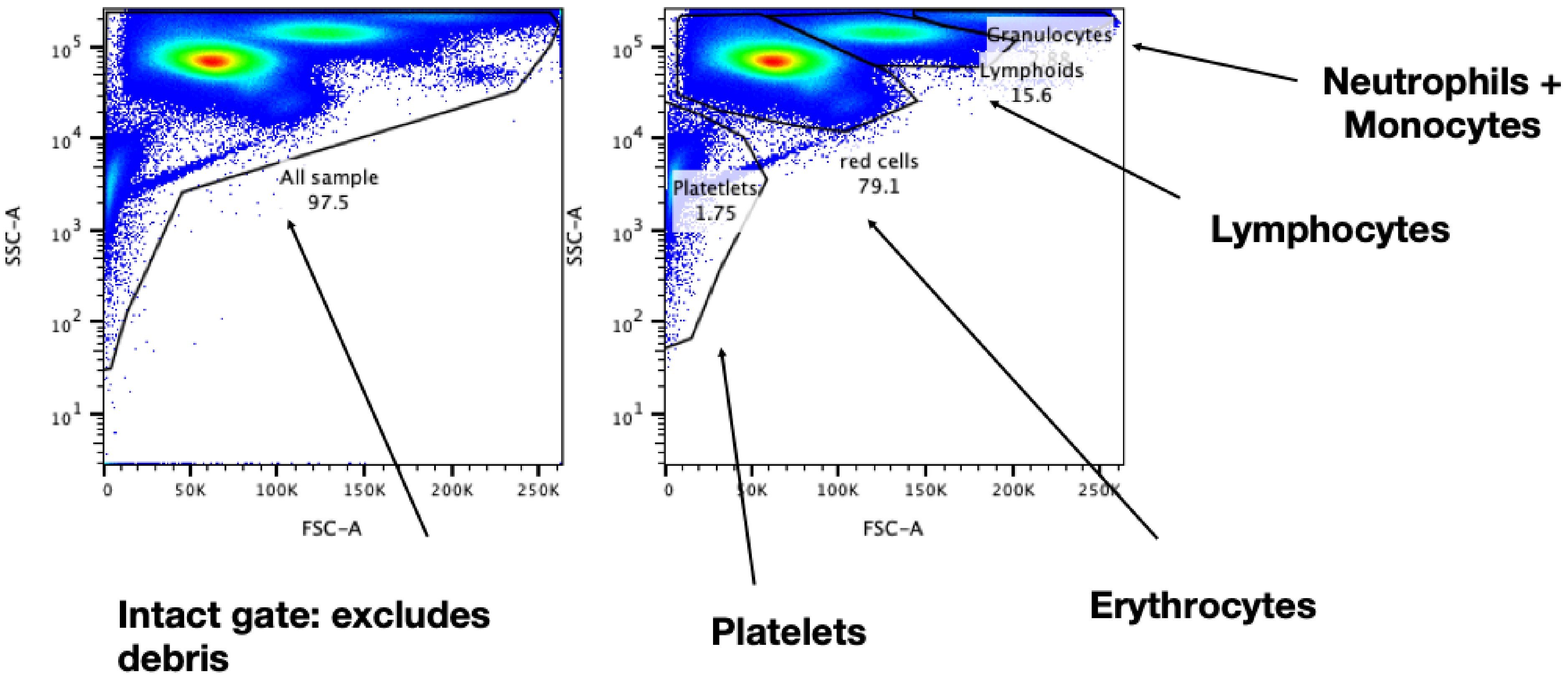

Erythrocytes - analysis based on intact gate and ab staining

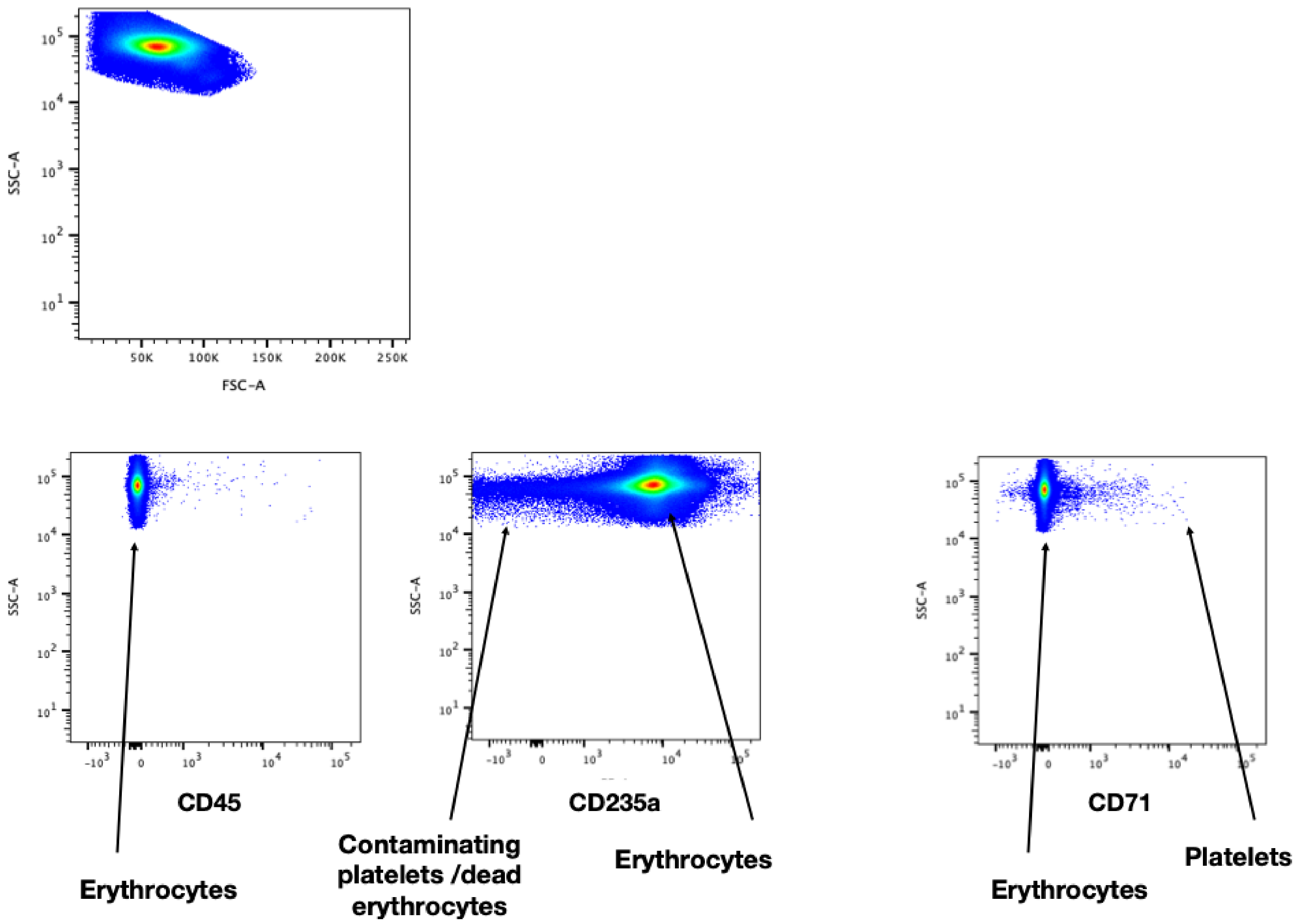

Platelets – analysis based on scatter gating and ab staining

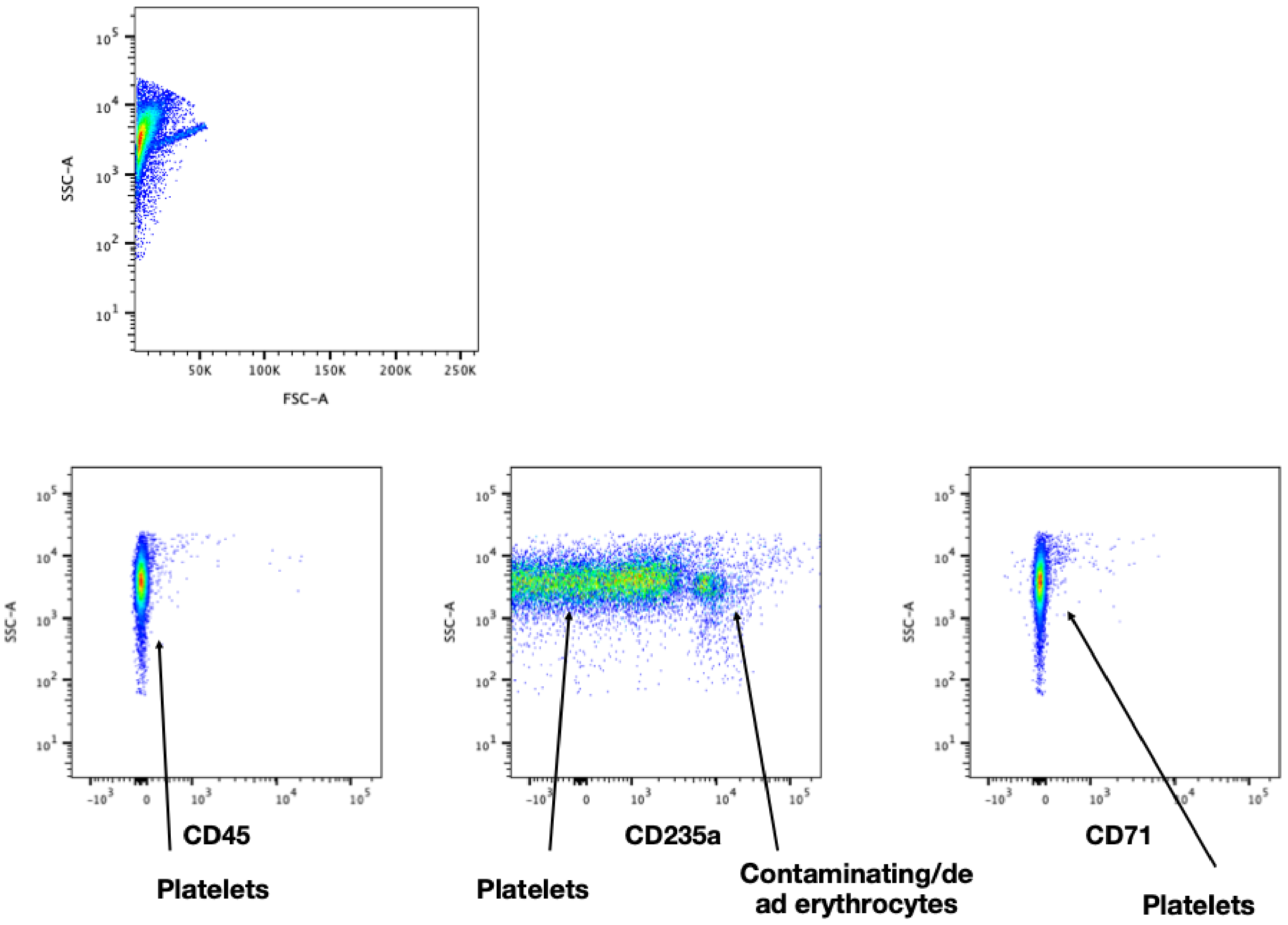

Supplement: Supplementary file 1 [file DataSheet1.pdf]
